# Selectivity for TP53 signalling drives the mode of action of a highly potent *N*,*O*,*O*-tridentate naphthoquinone-based organo-ruthenium anticancer drug candidate†

**DOI:** 10.1039/d5sc00735f

**Published:** 2025-07-14

**Authors:** Alexander Rosner, Lukas Skos, Theresa Mendrina, Dina Baier, Michaela Hejl, Yasmin Borutzki, Mathias Gradl, Heiko Geisler, Thomas Mohr, Anton Legin, Michael A. Jakupec, Andrea Bileck, Christopher Gerner, Gunda Koellensperger, Petra Heffeter, Walter Berger, Bernhard K. Keppler, Wolfgang Kandioller, Samuel M. Meier-Menches

**Affiliations:** a Institute of Inorganic Chemistry, University of Vienna Waehringer Str. 42 Vienna 1090 Austria samuel.meier-menches@univie.ac.at wolfgang.kandioller@univie.ac.at; b Doctoral School in Chemistry, University of Vienna Waehringer Str. 38 Vienna 1090 Austria; c Department of Analytical Chemistry, University of Vienna Waehringer Str. 38 Vienna 1090 Austria; d Center for Cancer Research and Comprehensive Cancer Center, Medical University Vienna Borschkegasse 8a Vienna 1090 Austria; e Research Cluster “Translational Cancer Therapy Research” Vienna 1090 Austria; f Joint Metabolome Facility, University of Vienna and Medical University of Vienna Waehringer Str. 38 Vienna 1090 Austria

## Abstract

The metallodrug candidate [(3-ethyl-4-oxo-(pyrazolyl)-dihydronaphthalene)(cymene)ruthenium(ii)] (1a) was recently shown to exhibit exceptional antiproliferative activity towards the chemo-resistant SW480 cancer cell line with nanomolar potency. This study was conducted to elucidate the determining parameters of the mode of action of this *N*,*O*,*O*-tridentate organoruthenium compound *in vitro* and *in vivo*. Four metal(arenes) based on 3-ethyl naphthoquinone (3-Et-NQ, a) and 3-morpholine naphthoquinone (3-Morph-NQ, b) with ruthenium (1) and osmium (2) were synthesized and characterized. The 3-Morph-NQ ligand increased the solubility of the complexes, but showed a 30-fold reduction in antiproliferative activity compared to the 3-Et-NQ ligand and its complexes served as biologically inactive analogues. The solution reactivity of the four compounds was ligand- and metal-dependent, but they all showed selectivity for amino acids over nucleotides at biologically relevant concentrations. Drug effects were elucidated by proteome profiling at subcellular resolution and showed a pronounced ligand-dependent impact. The 3-Et-NQ containing ruthenium- and osmium(arenes) down-regulated TP53 as a central hub in the perturbation network, connected to down-regulated proliferative MAPK3 signalling. Complex 1a strongly down-regulated TP53 and potently inhibited cell cycle progression at the G2/M phase. Furthermore, 1a was found to disrupt the TP53-DDX3X-p21 signalling axis by direct interaction with DDX3X and loss of p21 expression. The 3-Et-NQ complexes, particularly 1a, showed tumour inhibitory effects *in vivo* in a CT26 colon carcinoma mouse model, while the 3-Morph-NQ complexes were inactive. Tissue proteome analyses of livers of 1a-treated mice displayed similar stress responses as observed *in vitro*. Finally, tumour tissue of 1a-treated mice revealed down-regulated EGFR, consistent with the impact on the TP53 signalling axis *in vitro*.

## Abstract 2

The mode of action of an *N*,*O*,*O*-tridentate organoruthenium complex based on a naphthoquinone backbone is found to selectively impact on the TP53-DDX3X-p21 signalling axis, which also explains its tumour inhibition *in vivo*.

## Graphical abstract

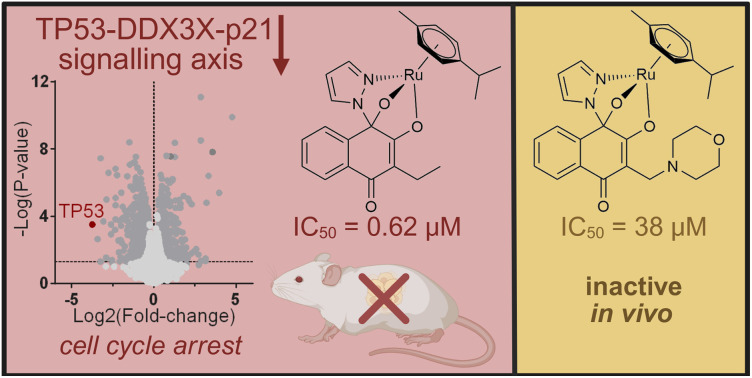

## 1. Introduction

The clinical success of the platinum anticancer agents cisplatin, carboplatin and oxaliplatin in conjunction with the DNA-targeting paradigm fuelled research efforts in the field of metals in medicine over decades.^1,2^ Gradual accumulation of experimental evidence, however, confirmed that the spectrum of biological effects of non-platinum metal-based anticancer drugs and drug candidates is more diverse and includes protein or metabolic targets.^3–6^ Molecular profiling technologies, *e.g.*, transcriptomics^7^ and (metallo-)proteomics,^8,9^ contributed towards a recent trend in mechanism-driven (metallo-)drug discovery.^6,10,11^ For example, based on transcriptomic studies, it was shown that the mode of action of oxaliplatin involves interference with ribosome biogenesis rather than direct DNA platination,^12^ which rationalizes its use in adjuvant chemotherapy of stage III colon cancer.^13^

**Fig. 1:**
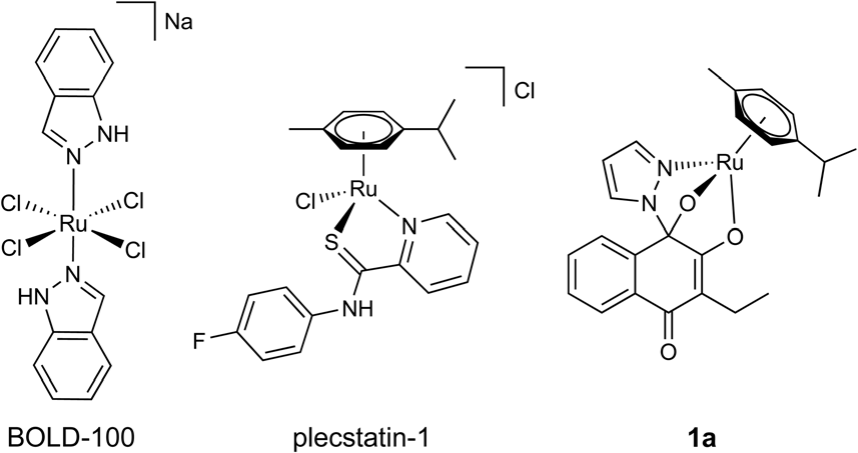
Chemical structures of the clinically investigated ruthenium(iii) complex BOLD-100 (left), and the investigational organoruthenium(ii) derivatives plecstatin-1 (middle) and 1a (right).

Ruthenium-based anticancer candidate drugs are among the most advanced classes of non-platinum therapeutics.^2,14^ This is highlighted by two representatives that are in clinical stages of investigation: first, BOLD-100 (formerly KP1339, IT-139, Fig. 1),^15^ a ruthenium(iii) complex, is currently evaluated in a phase Ib/IIa study against gastrointestinal tumours and features promising interim clinical outcomes (NCT04421820).^16,17^ Preclinical investigations on the mode of action (MoA) revealed a multimodal profile,^18^ including modulation of GRP78 with endoplasmic reticulum (ER) stress induction, accompanied by ribosomal interactions.^19^ Alterations in lipid metabolism were recently discovered^20^ and revealed that glucose-deprived cancer cells showed an enhanced vulnerability towards BOLD-100 treatment.^21^ Second, the ruthenium(ii)-based complex TLD-1433 was employed in the treatment of bladder cancer using photodynamic therapy^22,23^ and is currently undergoing a promising phase II study (NCT03945162).^24–26^

Pioneered by the groups of Sadler^27^ and Dyson,^28^ the family of organometallic ruthenium(ii) and osmium(ii) arene complexes is widely investigated.^14^ Typically, this metal(arene) motif is a reactive moiety that can be fine-tuned by versatile ligand systems, which in turn affect the antiproliferative properties of the complexes.^14,29–32^ Mono- and bidentate ligands are employed that are inert with respect to ligand exchange, while the remaining ligand sites are occupied by labile halido leaving groups. Such metal(arene) compounds are prodrugs, where the halido site undergoes ligand exchange reactions, *e.g.*, by aquation. This activates the prodrug, which can subsequently react with molecular targets by forming a coordination bond.^6,14,32,33^ Numerous studies reported on the metalation of biological nucleophiles by metallodrugs in cell-free studies,^34–36^ while the selectivity of these agents for certain nucleophiles is more challenging to investigate in a biological context.^37^ The nucleosome core particle was among the first relevant model systems to evaluate the binding selectivity of metal(arenes) towards nucleotides or amino acids.^3^ Interestingly, it was shown that ligand variation in ruthenium(arenes) would dictate the DNA *versus* protein reactivity and selectivity. Later, using a chemoproteomic approach, the ruthenium(arene) compound plecstatin-1 (Fig. 1) was found to exhibit unprecedented selectivity towards plectin in the cellular context.^38^ Modulation of plectin by plecstatin-1 induced distinct phenotypes that mirrored genetic plectin knockout,^39^ and shows promising antitumour activity against hepatocellular carcinoma.^40^ Furthermore, activation and reactivity by coordination was also found to be crucial for mediating the interaction of plecstatin-1 to plectin.^41^ Amongst others, an organo-osmium compound was found to exploit a metabolic vulnerability of mutated complex I in cellular respiration of a cancer cell line by a metabolic shift from glycolysis to oxidative phosphorylation.^42^

Consequently, it seems that the choice of the ligand scaffold strongly determines the biological effects of metal(arenes).^14^ The substitution of halido leaving groups of metal(arenes) with inert oxalato ligands retained cytotoxic potency in some cases,^43^ whereas inert tridentate ligand systems were found to reduce the cytotoxic potency,^44,45^ although some exceptions to this rule exist.^46^ This indicates that the formation of a coordination bond between the metal(arene) and a molecular target is generally necessary to obtain cytotoxic metal(arenes) and underlines the importance of ligand exchange kinetics for this family of metal-based anticancer candidate drugs.

Some of us recently reported on ruthenium(arene) derivatives based on a novel tridentate *N*,*O*,*O*-ligand system, which were formed *in situ* from bioactive hydroxy-1,4-naphthoquinones and 1,2-diazoles.^47^ Interestingly, these compounds, of which [(3-ethyl-4-oxo-(pyrazolyl)-dihydronaphthalene)(cymene)ruthenium(ii)] (1a, Fig. 1) is a promising representative, exhibited exceptional antiproliferative potency in the nanomolar range in a rather chemo-resistant colon carcinoma cell line,^47,48^ where the hydroxy-1,4-naphthoquinone ligands were largely inactive (showing half-maximal growth inhibition (IC_50_) at >100 μM). Although naphthoquinones are known inhibitors of NAD(P)H:quinone oxidoreductase 1 (NQO1),^49^ their organometallic complexes only moderately inhibited NQO1 relative to their cytotoxic potency suggesting that the potency of 1a is governed by the intact complex.^50^ It was then also shown that these compounds neither interacted with DNA nor produced reactive oxygen species (ROS).^48^ These findings led us to hypothesize that the compounds must follow a specific MoA independent of NQO1 inhibition that may explain their excellent potency *in vitro*. Here, we investigated the antiproliferative and tumour-inhibiting effects of highly potent metal(arenes) based on *N*,*O*,*O*-tridentate ligand systems to elucidate their MoA *in vitro* and *in vivo*. For this purpose, we synthesized a small panel of structurally related naphthoquinone-containing metal(arenes) and investigated their stability, as well as solution reactivity. We further evaluated structure–activity relationships (SARs) on the level of the proteome *in vitro* at a subcellular resolution and mechanistically confirmed specific effects on the TP53 signalling axis, which also seemed to manifest *in vivo*.

## 2. Results and discussion

### 2.1 Synthesis and characterization

**Fig. 2:**
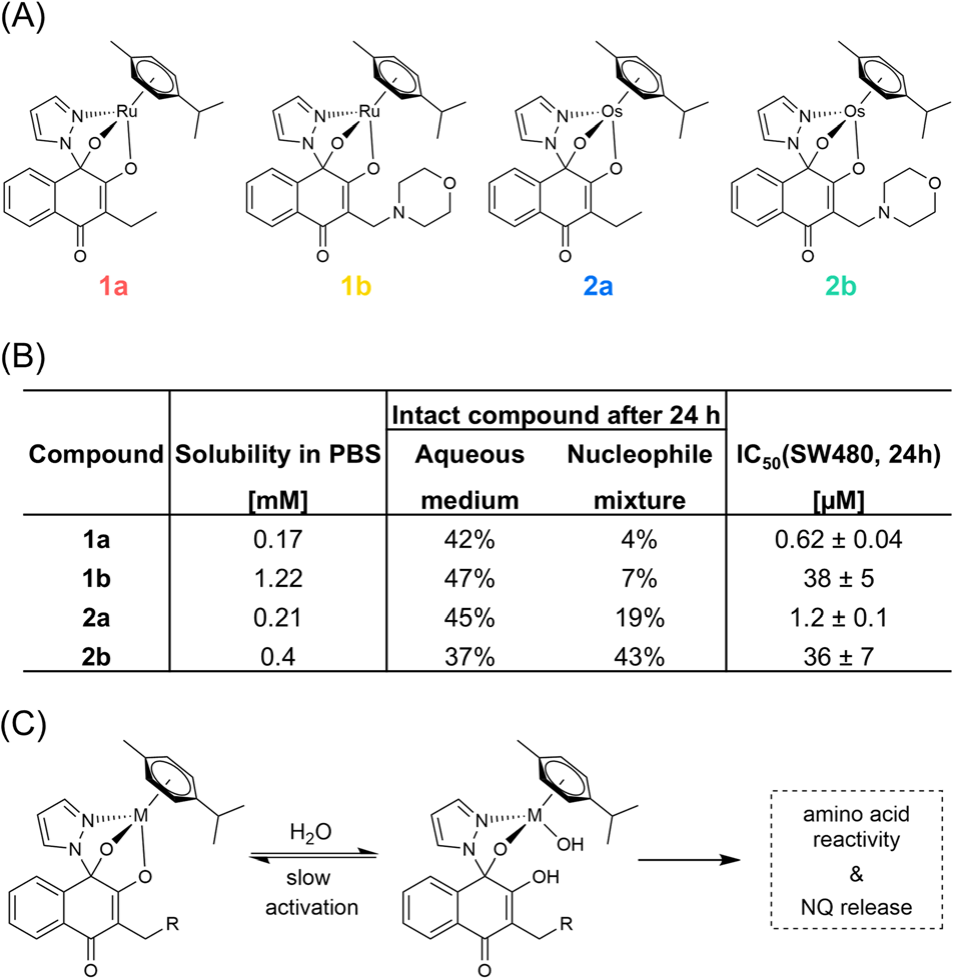
(A) Chemical structures of the investigated naphthoquinone-based metal(arenes) in this study. (B) Table highlighting solubility in phosphate buffered saline (PBS, with 1% DMSO), percentage of intact compound remaining after 24 h in aqueous medium and in the presence of the biological nucleophiles cysteine, methionine, histidine, adenosine triphosphate and guanosine triphosphate, each at 50 μM as revealed by mass spectrometry. Concentration of 50% growth inhibition (IC_50_) is displayed in the SW480 colon carcinoma cell line after 24 h incubation. (C) The scheme shows the proposed reactivity of the *N*,*O*,*O*-tridentate metal(arenes).

A small panel of structurally related *N*,*O*,*O*-tridentate metal(arenes) was synthesized to derive structure–activity relationships with respect to reactivity and biological activity. Starting from 3-ethyl naphthoquinone (3-Et-NQ, a) ruthenium(arene) lead compound 1a and its osmium analogue 2a were synthesized according to recently reported literature (Fig. 2A).^48^ To increase the solubility of the complexes, 3-morpholine naphthoquinone (3-Morph-NQ, b) was prepared and coordinated to the respective organometallic fragments yielding complexes 1b and 2b. The complexation reactions were performed in a one-pot microwave-assisted reaction in the presence of pyrazole, the NQ ligand, the dimeric metal(arene) precursor and triethylamine, to obtain the neutral *N*,*O*,*O*-tridentate-based organometallics. The complexes were obtained in good yields and were characterized by nuclear magnetic resonance (NMR), mass spectrometry (MS) and elemental analysis (ESI, Fig. S1–S9†). Formation of the desired complexes was confirmed by ^1^H- and ^13^C-NMR spectroscopy. The OH proton of the free ligand vanished upon coordination and furthermore, the arene signals were found as four distinguishable doublets, which indicated successful coordination of the NQ ligand. Formation of the expected tridentate complexes could be unambiguously confirmed by the presence of the hemiaminal carbon at around 100 ppm in the corresponding ^13^C-NMR spectra (ESI, Fig. S2–S5†). Proton and sodium adducts of the respective organometallics were found in HR-MS studies and the observed isotopic patterns are in good agreement for mononuclear Ru species (ESI, Fig. S6–S9†). Elemental analyses revealed the hygroscopic nature of the compounds due to the increased O-content in all complex samples. The obtained data confirmed sufficient purity for further biological examinations. As expected, the introduction of the 3-Morph-NQ fragment remarkably increased the solubility of the complexes by a factor of 2–7 compared to their 3-Et-analogues in phosphate buffered saline (PBS, Fig. 2B).

### 2.2 Stability and reactivity

These *N*,*O*,*O*-tridentate complexes were previously found to be highly stable and featuring slow activation kinetics. Nevertheless, they are activated by hydrolysis of the most labile M–O bond (Fig. 2C).^48^ Further hydrolysis releases the NQ ligand, because the free hemiaminal-containing NQ is unstable and the presence of biological nucleophiles accelerated NQ release.^48^ Here, the reactivity of 1a towards biological nucleophiles was investigated by NMR, UV-vis spectroscopy and ESI-MS to assess concentration-dependent reactivity differences. Compound 1a was incubated in an equimolar ratio with 9-ethylguanine and methionine or cysteine in D_2_O/20% DMSO. The samples were analysed on a 500 MHz NMR spectrometer after 0 h, 1.5 h and 24 h. At 1 mM, 1a remained remarkably stable in both the methionine- and cysteine-containing samples over the incubation period and no effects on chemical shifts were observed (ESI, Fig. S10†). The aromatic proton of 9-ethylguanine was consistently found at 7.6 ppm.

In contrast to the NMR analysis, the compounds were diluted to 40 μM and 80 μM for 1a/2a and 1b/2b, respectively and their stability was assessed by UV-vis spectroscopy continuously over 96 h (ESI, Fig. S11†). Under these biologically more relevant concentrations, the compounds showed ligand exchange reactions with distinct isosbestic points. While the ruthenium derivatives 1a/1b hydrolysed more rapidly over 24 h (1a 75%, 1b 76%, 2a 85%, 2b 94% of intact complex), the ethyl-NQ-containing organometallics 1a/2a seem to hydrolyse more extensively over 96 h (1a 53%, 2a 61%, 1b 64%, 2b 84% of intact complex).

In order to elucidate the transformation products of hydrolysis at micromolar concentrations, the four compounds were analogously incubated at 50 μM and analysed by ESI-MS^34^ in a time-dependent manner after 1.5 h, 24 h and 72 h. The four complexes of this series were quite stable over 24 h (ESI, Fig. S12†), but gradually released the NQ-ligands over the course of 72 h, which was observed by the formation of characteristic metal(cymene)-dimers containing pyrazole as bridging ligands. In addition, the observation of the free 3-Morph-NQ ligand after 24 h further supported the previously established activation mechanism (ESI, Fig. S12†). A mass signal, which was probably generated by in-source fragmentation corresponded to pyrazole-release. Ruthenium- and osmium organometallics behaved similarly, although osmium derivatives showed slower hydrolysis kinetics (ESI, Fig. S12†). In general, the intensity of the intact compounds after 24 h at 50 μM corresponded to approximately 40% of the total intensity of the mass spectrum in all four cases, not accounting for ionization efficiency (Fig. 2B).

Then, the compounds were analogously incubated with an equimolar mixture of cysteine, methionine, histidine, adenosine triphosphate and guanosine triphosphate to verify coordination preferences for amino acids over nucleotides. The reactivity of the four compounds was qualitatively similar, characterized by release of the NQ ligands and formation of mainly methionine and histidine-adducts with the metal(arene) (ESI, Fig. S13†). No metalation of the nucleotides was observed. In contrast to other *O*,*O*-ligand systems, which are typically deactivated within minutes by ligand release under such conditions,^51,52^ the *N*,*O*,*O*-tridentate organometallics studied here were characterized by increased stability. Nonetheless, 1a and 1b reacted nearly quantitatively within 24 h, while the Os analogues reacted at a slower rate so that intact 2a and 2b were still observable at 19% and 43% after 24 h, respectively (Fig. 2B and ESI, Fig. S12†). Again, the 3-Morph-NQ ligand was detected upon release from the metal centre, but hydrolysis dimers were not observed in the presence of bionucleophiles (ESI, Fig. S13†). The reactivity of the compounds towards an equimolar concentration of the model protein ubiquitin was characterized by similar kinetic behaviours and reactivity (ESI, Fig. S14†).

As the NMR, UV-vis and MS investigations showed, the activation and reaction kinetics of the studied complexes were clearly concentration dependent. Interestingly, we observed a stronger impact of the metal than the ligand on kinetics of activation over 24 h and adduct formation in the presence of biological nucleophiles in the following order: 1a ≥ 1b > 2a > 2b. The slower ligand exchange kinetics of osmium(ii) – compared to ruthenium(ii)arenes – is a known phenomenon.^52,53^ It originates from osmium being a third-row transition metal and therefore, being more inert compared to the second-row transition metal ruthenium. Indeed, isosteric osmium organometallics often exhibit longer half-lives of hydrolysis.^29^

### 2.3 Antiproliferative activity *in vitro*

Compound 1a showed potent antiproliferative activity with an IC_50_-value of 0.046 ± 0.007 μM in the rather chemo-resistant SW480 colon cancer cell line over a 96 h incubation time (Table S1†).^48^ Interestingly, *N*,*O*,*O*-tridentate ruthenium(arenes) of this type were selectively active towards the SW480 cancer cell line,^48^ while the naphthoquinone ligands were previously found to be largely inactive *in vitro* (ESI, Table S1†).^48^ The antiproliferative activity of the four compounds 1a, 1b, 2a and 2b was then examined towards SW480 cells over a 24 h incubation time corresponding to the MoA studies below. The 3-Et-NQ complexes 1a and 2a showed IC_50_-values of 0.62 ± 0.04 μM and 1.2 ± 0.1 μM, respectively and the 3-Morph-NQ complexes 1b and 2b showed IC_50_-values of 38 ± 5 and 36 ± 7 μM, respectively (Fig. 2B, ESI, Fig. S15 and Table S1†). Thus, the ethyl derivatives 1a and 2a were >30 times more potent compared to their morpholine-analogues 1b and 2b, which showed only moderate cytotoxicity and serve as biologically inactive analogues. Moreover, the ruthenium-derivative 1a was slightly more potent than the osmium analogue 2a. Such an effect was not observed for the morpholine-derived compounds. It was previously shown that IC_50_-values of representatives of this compound family based on a 24 h incubation featured lower potency compared to a 96 h incubation.^50^ Nonetheless, the 3-Et-NQ complexes investigated here retained significant antiproliferative activity even after an incubation time of 24 h. This supported the fact that they do not exert their biological effect *via* direct DNA damage, but might involve other mechanisms. Furthermore, the pronounced antiproliferative activity of the 3-Et-NQ complexes must be due to specific mechanistic aspects, which were not perturbed by the 3-Morph-NQ complexes. This motivated the proteomics experiments detailed below.

### 2.4 Proteome-wide effects in SW480 cancer cells

**Fig. 3:**
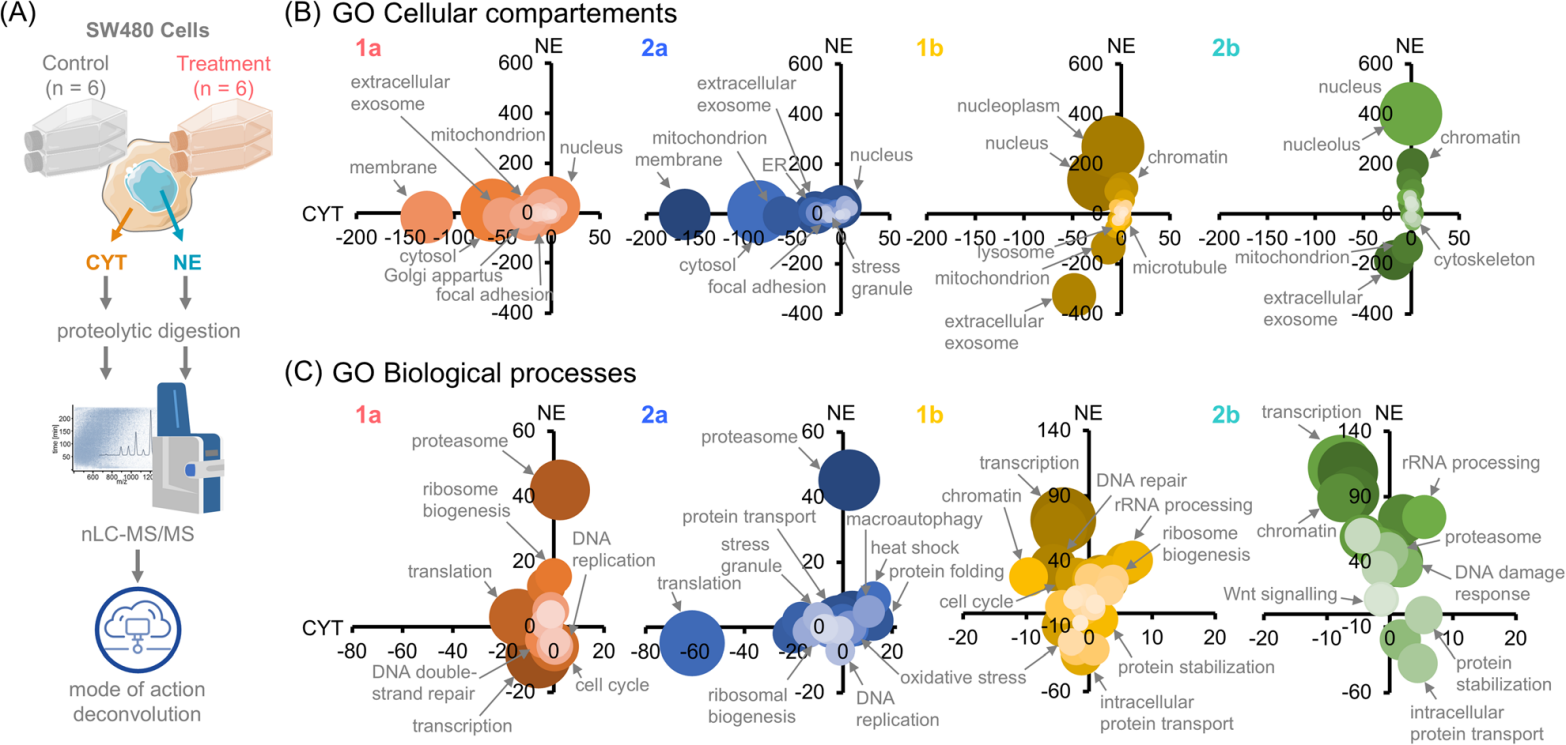
(A) SW480 cell populations were treated in hexuplicates. Cytoplasmic (CYT) and nuclear (NE) protein fractions were obtained. Samples were proteolytically digested and analysed by nano-liquid chromatography tandem mass spectrometry (nLC-MS/MS) in a data-dependent analysis mode. Proteomic perturbation data were then used for mode of action deconvolution. Regulomes of individual treatments are shown where each protein group was obtained from a term enrichment analysis according to gene ontology (GO) cellular compartments (B) or biological processes (C). The size of the bubbles correlates with the number of proteins in the group.

To gain insight into potential modes of action and derive structure–activity relationships on a molecular level, proteomic profiling experiments were carried out based on label-free quantification (LFQ) by nano-liquid chromatography tandem mass spectrometry (nLC-MS/MS). SW480 cancer cells were treated for 24 h with each compound at concentrations corresponding approximately to their individual IC_50_ values. Six replicates were used per condition. The perturbations were evaluated at subcellular resolution by fractionating cells into cytoplasmic (CYT) and nuclear (NE) protein fractions. Those have been separately proteolytically digested and analysed (Fig. 3A). The perturbations with the 3-Et-NQ and 3-Morph-NQ complexes were performed against their respective solvent-treated controls (CON), yielding a total of 72 samples. Scatter plots and principal component analyses of the samples revealed good precision of the method, as well as a strong and largely homogeneous impact of the treatments according to the respective conditions (ESI, Fig. S16†). The principle component analysis further revealed that 1b and 2b predominantly affected the nuclear fraction of the proteome (ESI, Fig. S16B†). A total of 5657 proteins were identified under all conditions combined. Statistical significance of individual protein regulations was calculated by multiple testing-corrected *P*-values based on 5% false discovery rate (FDR = 0.05 and *S*_0_ = 0.1). With respect to the number of significantly regulated proteins, 1a and 2a affected more strongly the cytoplasmic fraction, while 1b and 2b showed a predominant impact on nuclear fractions, indicating that the ligand directs the global impact of the perturbation. The dynamic range of significantly regulated proteins was large, from 16 regulated proteins by 1b in CYT to 1773 regulated proteins by 2b in NE (ESI, Table S2†).

The significantly regulated proteins of each treatment were then categorized into functional groups based on significantly enriched terms according to gene ontology cellular compartments (GO CC), biological processes (GO BP), and KEGG pathways (Fig. 3B and C, ESI, Fig. S17 and Data S1†). The position of each group represents the summed fold-changes of the individual proteins in the group according to the subcellular space, *i.e.*, CYT and NE fractions, while the size represents the number of proteins in each group.

Categorizing regulated proteins according to GO CC terms provides a global overview of the effects of each perturbation on cellular compartments.^54^ Again, the ligand type had a major influence on the perturbation profiles, since compounds based on 3-Et-NQ and 3-Morph-NQ led to changes predominantly in the cytoplasmic and nuclear fraction, respectively. Moreover, the identities of the groups overlapped significantly in compounds containing the same ligand. Compounds 1a and 2a, bearing the 3-Et-NQ ligand, induced a significant down-regulation of groups corresponding to the membrane, cytosol, mitochondria, extracellular exosomes and focal adhesion (Fig. 3B). Treatment with the ruthenium complex 1a additionally resulted in the down-regulation of proteins related to the Golgi apparatus, while 2a was associated with a stress granule group. The 3-Morph-NQ complexes 1b and 2b induced an upregulation of nuclear and nucleolar proteins, chromatin-related proteins, but down-regulated mitochondrial and extracellular exosome proteins. In more detail, compound 1b specifically regulated microtubular and lysosomal proteins, while 2b modulated cytoskeletal proteins.

In the next step, the perturbations were categorized according to GO BP to reveal the impact of a compound on biological processes in treated SW480 cancer cells (Fig. 3C). GO BP terms represent molecular programs that an organism tries to achieve and span various levels of biological organization.^54^ In fact, the groups of the proteasome, rRNA processing and ribosomal biogenesis were observed in all four treatments. On the one hand, complexes 1a and 2a were characterized by an upregulation of proteasomal proteins in the nuclear fraction and down-regulation of translational proteins in the cytoplasm. Additionally, cell cycle proteins were also down-regulated in the nuclear fraction and stress granule assembly was specific for 1a and 2a. On the other hand, compounds 1b and 2b featured the upregulation of transcriptional, chromatin-associated and rRNA processing proteins, while proteins for intracellular transport were down-regulated in the nuclear fraction. Proteins associated with protein stabilization were slightly upregulated in the cytoplasmic fraction. Compounds 1b and 2b had 13 common BP groups, among which were additionally DNA damage response, mRNA splicing and regulation of the apoptotic process. Interestingly, the cell cycle and mRNA transport groups were specific for the ruthenium(arene), while there were no specific groups for the osmium(arene) moiety.

The KEGG pathway analysis revealed quite compound-specific perturbations, but also showed the proteasome as a commonly upregulated pathway in the nuclear fraction (ESI, Fig. S17†). Overall, these data revealed detailed information about the impact of four organometallics on SW480 cancer cells and enabled the derivation of ligand-specific, metal-specific and compound-specific drug effects with relevance to their MoA.

Next, we investigated shared proteins across the treatments. Notably, despite the similarities of the functional groups in the regulomes, there were only a few overlapping proteins regulated in all four treatments (ESI, Fig. S18A†). The shared upregulated proteins in CYT and NE were searched using STRING,^55^ which revealed a pronounced proteasomal cluster, stemming from the nuclear fraction (ESI, Fig. S18B†). This was connected to the sole shared upregulated protein, sequestosome-1 (SQSTM1), which is known to have cytoprotective effects by acting as a bridge between ubiquitinylated proteins and autophagosomes.^56^ The proteasome was also among the few shared protein groups in the regulome (Fig. 3C). Additionally, we also observed the chaperone heat shock 70 kDa protein 1A (HSPA1A) upregulated in all treatments. Interestingly, there were no clusters among the shared down-regulated proteins, supporting the surprising fact that there is only a low overlap of regulated proteins in this panel of structurally similar organometallics (ESI, Fig. S18B†). Notable commonly down-regulated proteins include Prolow-density lipoprotein receptor-related protein 1 (LRP1) and basal cell adhesion molecule (BCAM). The former plays a role in endocytosis^57^ and the latter in cell adhesion and motility.^58^ There was also only a small number of shared regulated proteins when considering the same metal(arene) scaffold with the different ligands, but there was some overlap of the ruthenium- and osmium(arenes) bearing the same ligand. This highlighted again the stronger impact of the ligand on cellular responses on the proteome level compared to the metal(arene). This correlated with the IC_50_ values of the respective ligand systems, where the ethyl- and morpholine-derivatives dictated the antiproliferative potency irrespective of the metal(arene). NQO1, the target of the naphthoquinone ligands, was not regulated by any of the compounds investigated here (ESI, Fig. S19†).

### 2.5 Specific effects of the 3-Et-NQ complexes

To investigate molecular effects that might be responsible for the increased potency of the 3-Et-NQ complexes compared to the 3-Morph-NQ complexes, a STRING network analysis was performed with the shared regulated proteins only between 1a and 2a (ESI, Fig. S18†). Among the upregulated proteins, representatives of cellular stress responses were found, *e.g.*, NRF2 targets, heat shock response and cytoplasmic stress granules (ESI, Fig. S20A†). The NRF2-associated proteins were heme oxygenase 1 (HMOX1), glutamate-cysteine ligase regulatory subunit (GCLM) and oxidative stress-induced growth inhibitor 1 (OSGIN1). The heat shock response included HSPA1A and the ER chaperone BiP (GRP78, HSPA5), while the induction of the former was a feature of all four complexes.

**Fig. 4:**
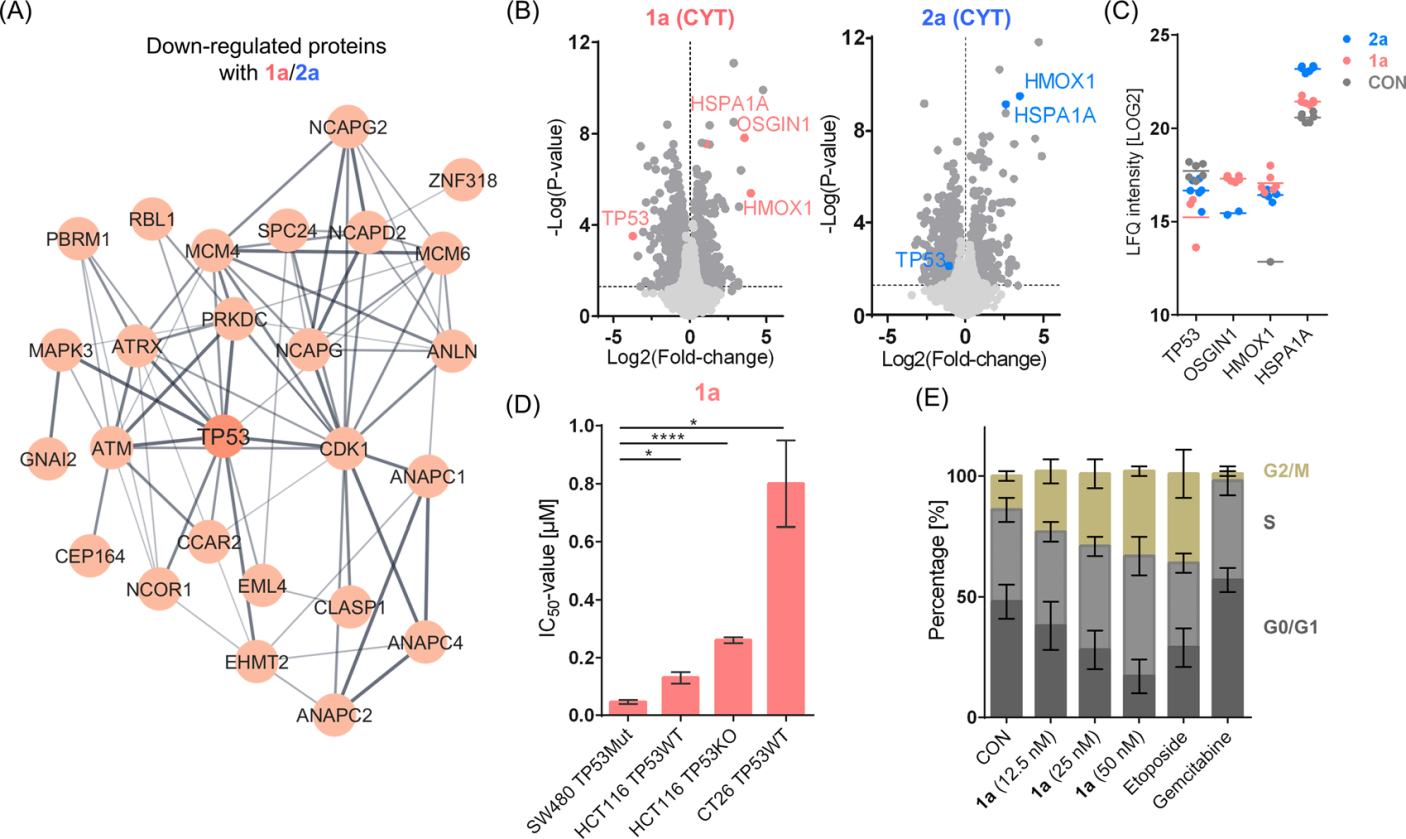
(A) STRING subnetwork of the shared 26 down-regulated proteins after treating SW480 cancer cells with 1a or 2a displaying the core cluster around TP53 related to cell cycle. (B) Volcano plots showing the proteome changes in the cytoplasmic (CYT) fractions of SW480 cells upon treatment with 1a and 2a. Proteins with statistically significant regulations are shown in dark grey (FDR = 0.05, *S*_0_ = 0.1). (C) Protein abundances in LOG2 space for selected proteins in control SW480 cells (CON) and upon treatment with either 1a or 2a. Six replicates were performed per condition. If not shown, proteins were not detected. (D) Bar chart of the IC_50_ values of 1a in cell lines with different TP53 status, *i.e.*, mutated (Mut), wild-type (WT) or knockout (KO). Cells were exposed for 96 h. Values are presented as means ± standard deviation. Significance was calculated by an unpaired two-tailed *t* test with Welch's correction: *p-value <0.05, ****p-value<0.0001. (E) Cell cycle analysis of SW480 cancer cells treated with 1a for 24 h at the indicated concentrations. Etoposide (100 nM) and gemcitabine (10 nM) served as controls for G2/M and G0/G1 arrest, respectively. Values are presented as means ± standard deviation.

The downregulated proteins featured an extensive network covering cell cycle, translation, mitochondrial respiration and extracellular matrix interactions (ESI, Fig. S20B†). Interestingly, the cellular tumour antigen p53 (TP53) was found as a central hub in the cell cycle network (Fig. 4A), together with mitogen-activated protein kinase (MAPK3) and signal transducer and activator of transcription 6 (STAT6), suggesting that a TP53-mediated antiproliferative effect would be crucial for the activity of the 3-Et-NQ complexes. TP53 is a known tumour suppressor and regulates cell cycle, DNA repair and apoptosis.^59^ TP53 is mutated in many tumours.^60^ SW480 cancer cells contain high levels of a doubly mutated TP53, with gain-of-function properties. Strikingly, TP53 was the strongest down-regulated protein upon treatment of SW480 cancer cells with 1a in the cytoplasmic fraction (Fig. 4B), but not for 2a (Fig. 4C). This suggests that 1a induces a more potent response compared to 2a and can efficiently destabilize TP53 levels in SW480 cells.

### 2.6 Compound 1a is specifically active in SW480 cancer cells with mutated gain-of-function TP53

To verify the involvement of TP53 in the MoA of 1a, its cytotoxicity was examined using the HCT116 cell line, which contains wildtype TP53 (ref. 61) and for which a TP53-knock-out variant was available. Interestingly, the IC_50_ value of 1a in the HCT116 TP53 wild-type (TP53WT) was 0.13 ± 0.02 μM and thus, approximately four-fold less potent compared to the SW480 cells (Fig. 4D). Moreover, the HCT116 TP53-knockout (TP53KO) cell line exhibited an IC_50_ value of 0.26 ± 0.01 μM, further reducing the potency by a factor of two, underscoring the dependence of cytotoxic potency on TP53 status of 1a. The IC_50_ value of 1a in the murine CT26 colon carcinoma cells, containing wildtype TP53,^62^ was determined to be 0.80 ± 0.15 μM. Consequently, 1a was most active towards the TP53-mutated SW480 cell line. This cell line depends on TP53-mediated signalling for proliferation.^63^ TP53 controls the S and G2/M checkpoints of the cell cycle. Therefore, destabilizing TP53 would lead to an accumulation of cells in these phases of the cell cycle. SW480 cancer cells were treated with 1a at increasing concentrations from 12.5 to 50 nM for 24 h and the cell cycle distribution was assessed (Fig. 4E). A dose-dependent increase in the S and G2/M phases of the cell cycle was clearly observed in the range of the IC_50_. The potency of 1a treatment on cell cycle distribution was similar to the positive control etoposide for which a 100 nM treatment was applied, but contrasted to gemcitabine, which induced a G0/G1 arrest. Importantly, the topoisomerase II poison^64^ etoposide induces to DNA double strand breaks and a TP53-mediated DNA damage response.^65^

### 2.7 Compound 1a deregulates the TP53-DDX3X-p21 signalling axis

**Fig. 5:**
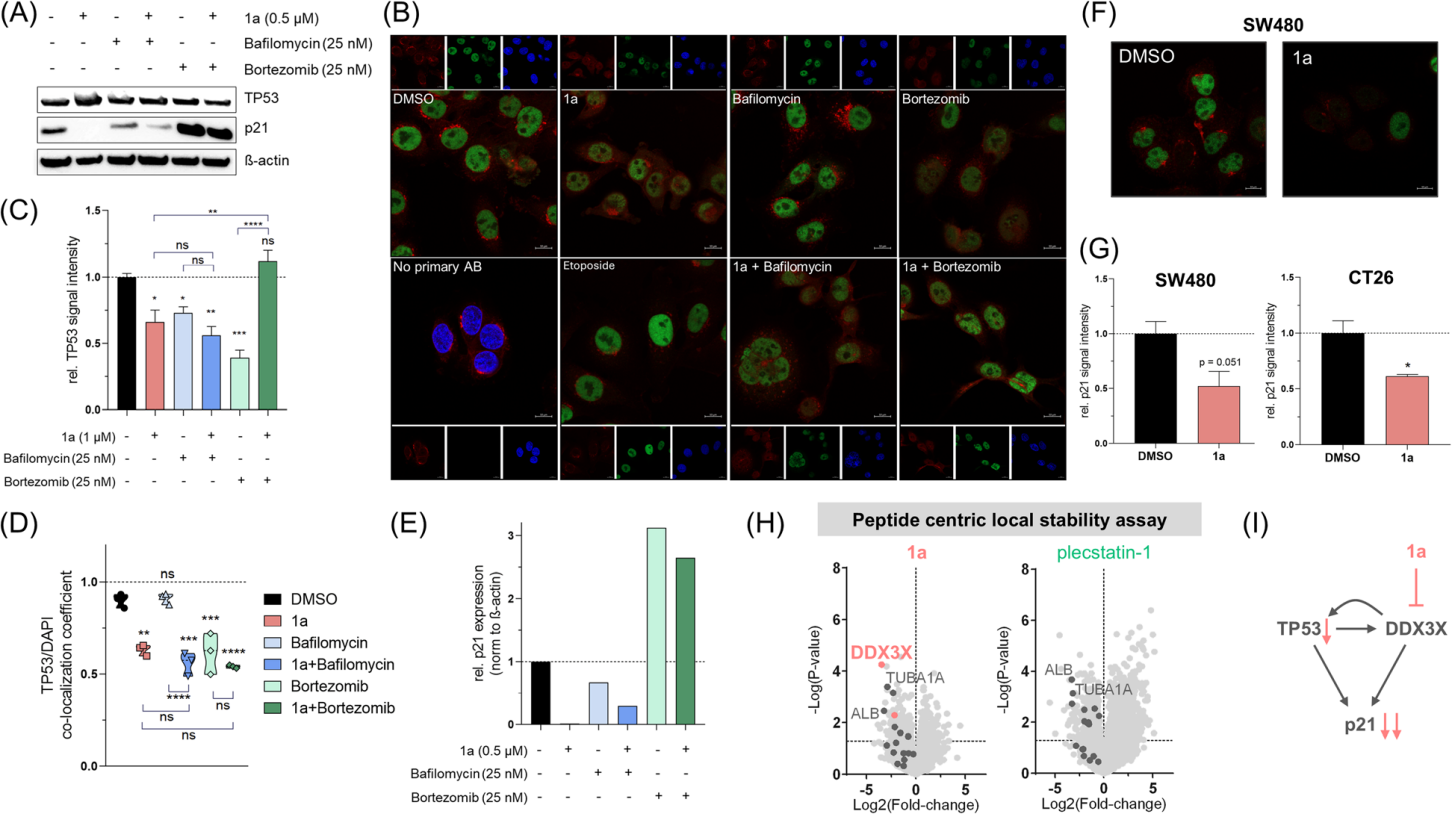
(A) Protein expression levels of TP53 and p21 in whole cell lysates of SW480 cells treated with indicated compounds for 24 h and analyzed by western blotting. β-Actin served as loading control. (B) Immunofluorescence staining of TP53 (green) in SW480 cells following treatment with 1 μM of 1a without or with 25 nM of bafilomycin or bortezomib compared to DMSO control or 100 nM of etoposide for 24 h. Cytoplasm and nuclei were stained using rhodamine-labelled wheat germ agglutinin (WGA, red) and DAPI (blue), respectively. (C) Mean TP53 signal intensity relative to DMSO control quantified from (B). One-way ANOVA with Tukey's multiple comparisons test: *p < 0.05, **p < 0.005, ***p < 0.0005, ****p < 0.0001. (D) TP53/DAPI co-localization coefficient calculated from (B). (E) p21 protein expression relative to DMSO control, normalized to β-actin quantified from (A) using Image J. (F) Immunofluorescence staining of p21 (green) in SW480 cells following treatment with 1a (1 μM) as compared to control for 24 h. Cytoplasm was stained using rhodamine-labelled WGA (red). (G) Quantification of p21 immunofluorescence signal following treatment with 1a (1 μM) as compared to control for 24 h in SW480 (F) and CT26 cells. Unpaired two-tailed Student's *t*-test: *p < 0.05. (H) Peptide centric local stability assay was performed to identify potential direct interaction partners of 1a and plecstatin-1, revealing DDX3X as a specific interactor of 1a. Each dot depicts a tryptic peptide. (I) Scheme of the proposed impact of 1a on the TP53-DDX3X-p21 signalling axis.

For further in-depth investigation, we assessed TP53 and downstream effector p21 expression. In whole cell lysates, no down-regulation of TP53 was observed upon 1a treatment (Fig. 5A). However, immunofluorescence staining revealed decisive loss of TP53 signal intensity (Fig. 5B and C), corroborating the findings from proteome profiling (*cf.*Fig. 4A and B). Again, etoposide was used as positive control for the induction of TP53 expression and indeed showed enhanced nuclear accumulation of TP53 (Fig. 5B). Additionally, changes of TP53 expression were assessed after incubation with subtoxic concentrations (ESI, Fig. S21†) of bafilomycin or bortezomib, which are inhibitors of lysosomal acidification and the proteasome, respectively, with the aim to reveal potential degradation routes of TP53. While both inhibitors on their own reduced TP53 signals, only combination with bortezomib induced accumulation of TP53 indicating the proteasome to be mainly responsible for TP53 degradation and turnover in SW480 cancer cells. Moreover, assessment of the TP53/DAPI co-localization coefficient revealed that 1a and bortezomib retained TP53 in the cytoplasm in a comparable manner (Fig. 5D). Drug combination did not further enhance this effect. Assessment of TP53 expression in CT26 cells revealed downregulation associated with proteasomal degradation pathway (ESI, Fig. S22†), in line with observations in SW480 cells. Growth arrest is induced by p21 upon p21 promoter transactivation by TP53.^66^ Consequently, p21 expression levels were also analysed upon treatment. Unexpectedly, 1a treatment significantly reduced p21 expression in SW480 and CT26 cells (Fig. 5A, E–G), which also seemed to be degraded *via* the proteasomal pathway (Fig. 5E).

A recently reported^67^ peptide centric local stability assay was then performed to determine whether 1a would directly interact with TP53 or p21. The method relies on a limited-proteolysis strategy, where ligand-binding on target proteins is expected to increase the stability of the proteins against proteolytic digestion. This specifically enables the identification of target proteins of small molecules. For this purpose, a SW480 whole cell lysate was treated with DMSO, 1a (1 μM) or plecstatin-1 (20 μM), each in four replicates (Fig. 5H). Being a ruthenium(arene) derivative, plecstatin-1 was used to assess potential non-specific interactors of the ruthenium(cymene) fragment. Among the identified 930 proteins from 2666 peptides for 1a, we did not detect TP53, or p21, probably due to the limited digestion efficiency. However, among the selective interaction partners, we found specific peptides of ATP-dependent RNA helicase DDX3X, tubulin alpha-1A (TUBA1A) and albumin (ALB). Several peptides of TUBA1A and ALB were also found to be stabilized by plecstatin-1 indicating low specificity. This results in DDX3X being the most probable direct binding partner of 1a among the subpopulation of identified proteins. Interestingly, DDX3X interacts with TP53 and can change the accumulation of TP53 in the cytoplasm and nucleus.^68^ It was demonstrated previously that the interaction of DDX3X with TP53 is dependent on the mutational status of the latter and moreover, p21 transcription is promoted by DDX3X *via* direct interaction with SP1 in a TP53-dependent^69^ and -independent manner.^70^ This, together with our data, suggests that 1a impairs TP53 functionality leading to p21 loss, which seems to be mediated by direct interaction of 1a with DDX3X (Fig. 5I) and might explain the exceptional potency of this compound towards the rather chemoresistant SW480 cancer cell line.

### 2.8 Anti-tumour activity in the CT26 mouse model

Tumour inhibition of the four compounds was evaluated in subcutaneous CT26 allografts using BALB/c mice, since it represents an appropriate immune competent mouse model to study colon carcinoma. The compounds were administered intraperitoneally (i.p.) over two weeks at doses of 10 mg kg^−1^ for the 3-Et-NQ complexes and 30 mg kg^−1^ for the 3-Morph-NQ complexes. The different dosages were selected as maximal tolerable doses (MTDs) after an initial toxicity screen (data not shown).

**Fig. 6:**
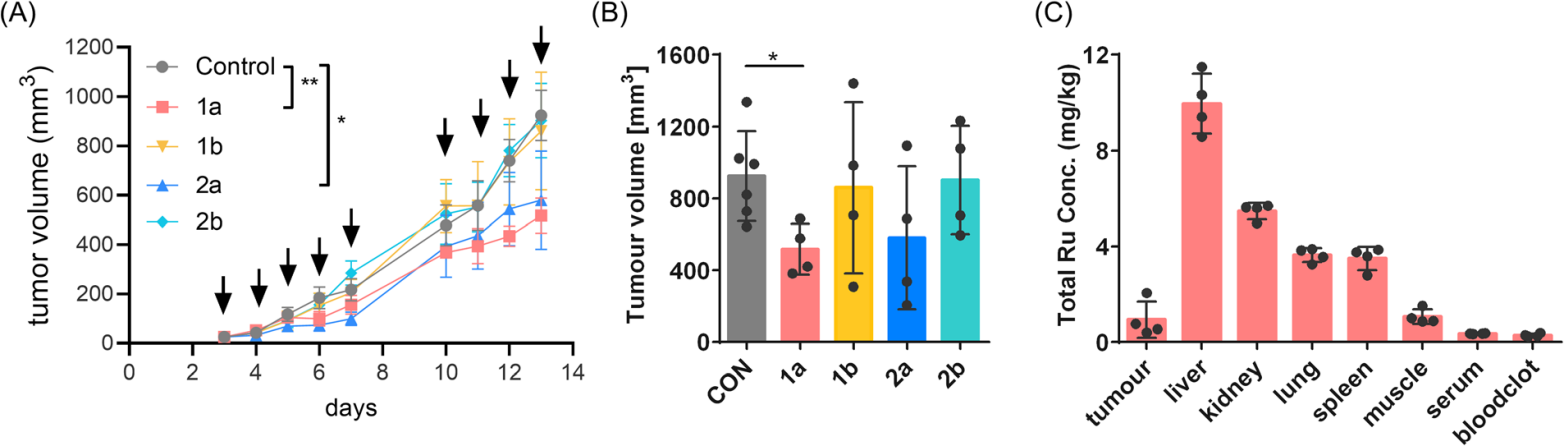
(A) Anticancer activity of the indicated compounds *in vivo*. CT26-bearing BALB/c mice were treated i.p. over two weeks (indicated by arrows) with 10 mg kg^−1^ of 3-Et-NQ complexes (1a and 2a) and with 30 mg kg^−1^ of 3-Morph-NQ complexes (1b and 2b). Data are presented as mean ± SEM. Statistical significance was tested by two-way ANOVA using Dunnett's multiple comparison test. *p < 0.05, **p < 0.01. (B) Bar chart showing the tumour volume at the last day in vehicle-treated controls and compound-treated mice. Bars represent mean values. Significance was calculated by an unpaired two-tailed *t* test with Welch's correction: *p-value <0.05. (C) Tumour, organs and blood were collected of 1a-treated mice and the total ^101^Ru content was quantified by inductively coupled plasma mass spectrometry. Bars represent mean values.

Compounds 1a and 2a were found to significantly inhibit tumour growth compared to vehicle-treated controls, while 1b and 2b were inactive (Fig. 6A). Consequently, the nature of the ligand not only determines *in vitro* activity, but also drives *in vivo* tumour inhibition. Compound 1a achieved significant tumour inhibition featuring a significant 44% reduction in tumour volume on day 13 (Fig. 6B).

Furthermore, tumour, organs and blood of the 1a-treated mice were collected 4 h after the last injection. The average metal content was quantified by inductively coupled plasma mass spectrometry of tissue homogenates using the ^101^Ru isotope. Intraperitoneally administered compounds are primarily absorbed through the portal circulation, which can lead to a substantial liver exposure.^71^ Indeed, 1a distributed to the liver, where a ^101^Ru content of 10 ± 1 mg kg^−1^ was detected (Fig. 6C). A smaller amount accumulated in the tumour, corresponding to 0.9 ± 0.7 mg kg^−1 101^Ru. This paralleled earlier findings of other metal(arenes) in this tumour model.^72^ Interestingly, 1a neither accumulated in blood serum nor in the blood clot with ^101^Ru contents consistently <0.4 mg kg^−1^.

### 2.9 Evaluation of drug effects *in vivo*

**Fig. 7:**
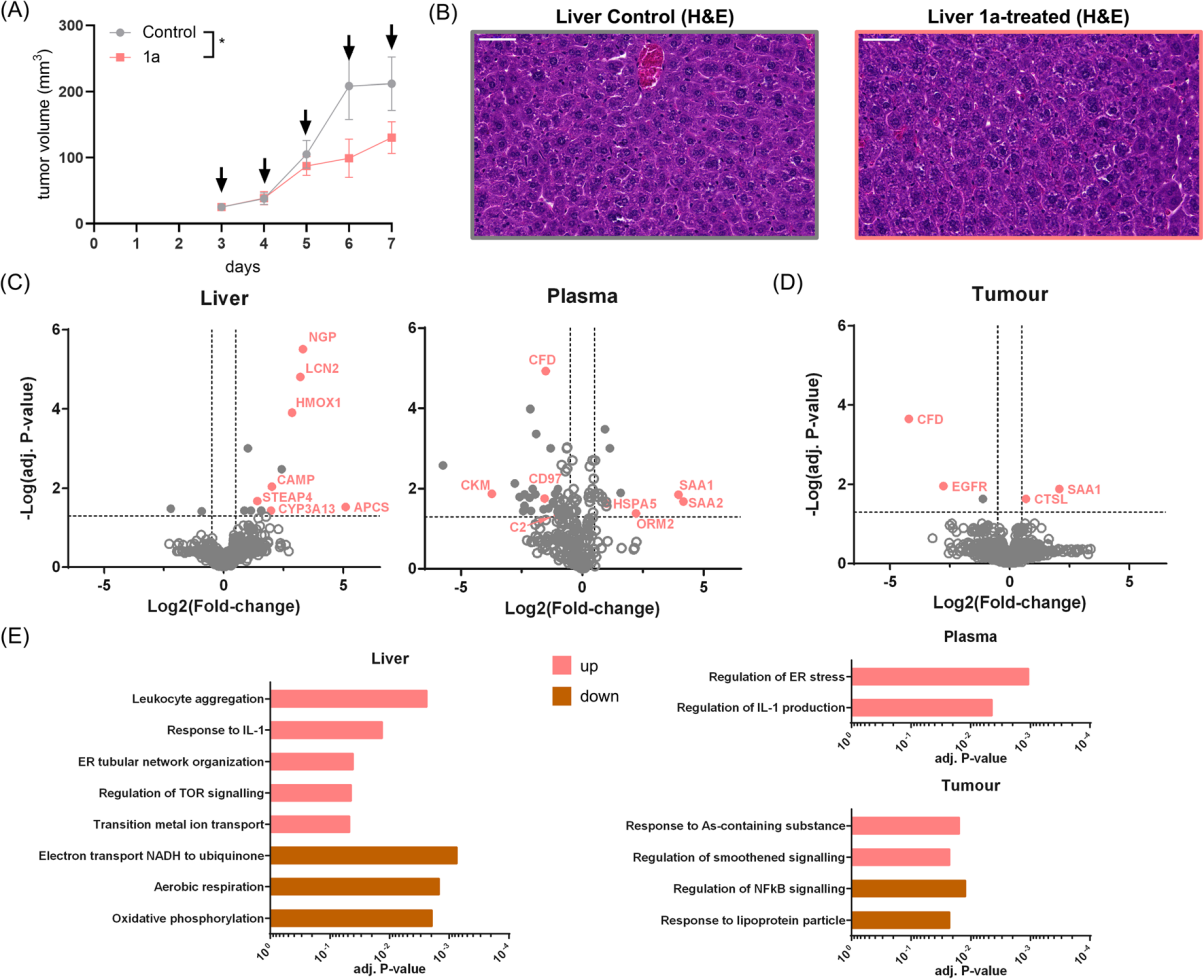
(A) Anticancer activity of 1a*in vivo*. CT26-bearing BALB/c mice were treated i.p. on five consecutive days (indicated by arrows) at a dose of 10 mg kg^−1^. Impact on tumour growth; data are presented as mean ± SEM. Statistical significance was tested using the mixed-effects model (REML): *p-value <0.05. Tumour, liver and blood plasma of these mice were collected and analysed by proteome profiling. (B) Representative microscopy images of liver tissue harvested from untreated and treated mice. Tissues were paraffin-embedded, followed by cutting and histological staining by hematoxylin and eosin (H&E). Pictures were taken using a Slidescanner (magnification: 40×; scale bar: 50 μm). (C) Volcano plots of the proteomic effects of 1a-treated *versus* vehicle-treated mice highlighting systemic effects in the liver and blood plasma. Statistical significance was calculated using LIMMA and a multiple-testing correction based on Benjamini–Hochberg. (D) Volcano plot of the proteomic effects of 1a-treated *versus* vehicle-treated mice highlighting local effects in the tumour. Statistical significance was calculated using LIMMA and a multiple-testing correction based on Benjamini–Hochberg. (E) Gene set variation analysis (GSVA) of the tissue proteomic data according to upregulated and down-regulated GO BPs. Bars represent adjusted *P*-values of statistically significant term enrichments.

The significant tumour-inhibition of 1a together with the selective impact on TP53-mediated signalling motivated investigations into *in vivo* drug effects. For this purpose, an additional 1-week experiment was performed by treating BALB/c mice bearing CT26 tumours with 1a administered at 10 mg kg^−1^ for five consecutive days. The experiment confirmed the statistically significant tumour inhibition of 1a (Fig. 7A). On day five, tumour, liver and blood were harvested two hours after the last injection of 1a. Blood plasma and livers were harvested to obtain insight into systemic drug effects and tumours were collected to obtain information about local effects of the candidate drug.

Liver tissue for histological assessment was fixed with formaldehyde and then paraffin-embedded. Haematoxylin and eosin (H&E) was used to stain tissue slices. Despite being the most exposed organ, liver sections of 1a-treated mice showed largely healthy physiology (Fig. 7B).

For proteomic workup, the fresh tissue samples were quickly rinsed with PBS and shock-frozen in liquid nitrogen. Blood plasma was immediately prepared by centrifugation of the collected blood in ethylenediaminetetraacetic acid (EDTA) tubes. The samples were analysed in technical duplicates due to the expected larger heterogeneity and statistical evaluation was performed using the LIMMA algorithm^73^ and gene set variation analysis (GSVA).^74^ A total of 2770 proteins were identified in liver with 14 statistically significantly regulated proteins and 285 proteins in plasma samples with 79 statistically significant regulations (Fig. 7C). Then, proteomic analysis revealed 3867 proteins in tumour samples of which 5 showed statistically significant regulations (Fig. 7D). Statistical significance was calculated based on multi-parameter testing-adjusted *P*-values ≥0.05. The distribution of 1a in CT26 tumour-bearing mice had revealed a significant accumulation in the liver and minor amounts in tumour tissue, suggesting a potential involvement of systemic contributions to its tumour-inhibiting effect.

Although the liver showed a normal physiology, the proteomic data evidenced the upregulation of detoxification enzymes in 1a-treated mice, *e.g.*, cytochrome P450 3A13 (CYP3A13), heme oxygenase 1 (HMOX1) and metalloreductase STEAP4 (STEAP4, Fig. 7C). As one of the most exposed organs to 1a, the upregulation of HMOX1 also correlated with the *in vitro* drug effects.

Moreover, the induction of lipocalin-2 (LCN2), neutrophil granule protein (NGP), serum amyloid *P*-component (APCS) and cathelicidin antimicrobial peptide (CAMP) indicated a drug-induced acute phase response in the liver. These effects were further supported by the statistically significant enrichment of GO BP terms in the GSVA (Fig. 6E and ESI, Table S3†), *e.g.*, “response to IL-1” and “leukocyte activation”. The upregulated term “transition metal ion transport” was indicative of metal transport, including iron homeostasis and “TOR signalling” related to a restriction of nutrients under this treatment challenge. This was complemented by a down-regulation of several terms related to mitochondrial respiration, which can accompany an acute phase response (Fig. 7E).^75,76^

Acute phase proteins were also found upregulated in blood plasma of 1a-treated mice (Fig. 7C). The liver-derived c-reactive protein (CRP), serum amyloid A (SAA1/SAA2), APCS and α_1_-acid glycoprotein 2 (ORM2) are classical acute phase markers in plasma. While CRP and SAA1/SAA2 are usually mediating pro-inflammatory processes,^77^ ORM2 has immunomodulatory properties and might dampen excessive inflammation reactions.^78^ Several complementary components were found to be down-regulated, including C2, C5, C8alpha-gamma and complement factor D (CFD). Similarly, GSVA in plasma revealed a term related to “regulation of IL-1 production”, involved in inflammatory and immune regulatory processes.

The most pronounced changes in the tumour mirrored those in plasma, including regulated SAA1 and CFD, which may represent a consequence of tumour vasculature, and do not directly represent 1a-treatment in the tumour (Fig. 7D). GSVA revealed a decreased “regulation of NFkB signalling” and an induction of “response to As-containing substance”. The latter included HMOX1, similarly to the observed effects in the liver and *in vitro* in SW480 cells. This is representative of the stress response induced by 1a at the tumour site. Moreover, EGFR was found to be down-regulated in 1a-treated mice. This receptor of the MAPK3 pathway is linked to TP53 and directly involved in proliferation signalling.^79,80^ Indeed, down-regulated EGFR was connected to down-regulated RHOA and ACTN4 signalling, as well as RELA (p65, NFkB) (ESI, Fig. S23†). Furthermore, DDX3X was shown to regulate EGFR in breast cancer.^81^ Down-regulation of EGFR *in vivo* might therefore reflect a further crucial direct effect of 1a targeting the TP53-DDX3X-p21 signalling axis as revealed from the *in vitro* experiments.

## 3. Conclusions

Metal(arenes) containing *N*,*O*,*O*-tridentate ligands, which were obtained *in situ* from dimeric metal precursors, diazoles and naphthoquinone ligands, showed exceptional antiproliferative activity in the nanomolar range, while the ligands alone were largely inactive. In contrast to other metal(arenes) containing bidentate ligands, this family is kinetically inert over a prolonged time period, but retains reactivity by hydrolysis of a metal–oxygen bond of the naphthoquinone ligand. Preferential binding of the complexes to amino acids over nucleotides was confirmed, accompanied by release of the naphthoquinone ligand from the complex. Among the four complexes, the ligand strongly determines the antiproliferative activity with the 3-ethyl naphthoquinone organometallics showing >30-fold higher potency compared to the 3-morpholino derivatives. Similarly, the ligand influenced protein perturbation profiles more strongly than the metal. The shared effects of the 3-ethyl naphthoquinone organometallics revealed TP53 as a central hub in the perturbation network, directly impacting on proliferative signalling. Furthermore, the mutant TP53 cell line SW480 seemed to be particularly sensitive to these compounds. The ruthenium lead compound considerably affected cell cycle progression at the G2/M phase, which was associated with disturbance of the TP53-DDX3X-p21 signalling axis and loss of p21 expression, probably mediated by direct modulation of DDX3X. The 3-ethyl naphthoquinone organometallics also showed anticancer activity in the CT26 mouse model, where especially the ruthenium(arene) derivative significantly reduced tumour size. Drug effects of the 3-ethyl naphthoquinone containing an organoruthenium complex were then assessed in the same mouse model by proteome profiling. To distinguish direct from indirect drug effects at the tumour site, systemic responses were considered. As a result, the down-regulated EGFR in the tumour was identified as the direct effect of treatment. EGFR levels were previously found to be controlled by TP53-DDX3X-p21 signalling. The strategy presented here can be used to elucidate the modes of action of phenotypically discovered metal-based anticancer drugs *in vitro* and *in vivo*. The antitumour activity of 1a*via* the TP53-DDX3X-p21 signalling axis and its dependence on the mutational status of TP53 warrants further mechanistic investigations.

## Author contributions

Conceptualization, P. H., S. M. M.-M. and W. K.; data curation, A. R., L. S., T. Me., T. Mo., A. B., P. H., M. A. J., S. M. and M.-M.; formal analysis, A. R., L. S., T. Me., H. G., T. Mo., and A. L.; funding acquisition, P. H., W. B., C. G., G. K., B. K., W. K., and S. M. M.-M.; investigation, A. R., L. S., T. Me., D. B., M. H., Y. B., M. G., H. G., A. B., and A. L.; methodology, P. H., S. M. M.-M. and W. K.; project administration, P. H., S. M. M.-M. and W. K.; resources, P. H., W. B., G. K., C. G., M. A. J., B. K., S. M. M.-M. and W. K.; supervision, P. H., W. B., G. K., C. G., M. A. J., S. M. M.-M. and W. K.; validation, D. B., P. H., W. B., G. K., C. G., M. A. J., S. M. M.-M. and W. K.; visualization, A. R., L. S., T. Me., D. B., A. L., and S. M. M.-M.; writing – original draft preparation, A. R., L. S., T. Me., M. A. J., P. H., W. K. and S. M. M.-M.; writing – review and editing, A. R., L. S., D. B., P. H., W. K. and S. M. M.-M.; all authors have read and agreed to the published version of the manuscript.

## Ethical statement

All experiments were carried out with male BALB/c mice according to the regulations of the Ethics Committee for the Care and Use of Laboratory Animals at the Medical University Vienna (BMWF-2022-0.770.291), following the guidelines from the Austrian Animal Science Association and the Federation of European Laboratory Animal Science Associations (FELASA).

## Conflicts of interest

There are no conflicts to declare.

## Supplementary Material

SC-016-D5SC00735F-s001

SC-016-D5SC00735F-s002

## Data Availability

Proteomic data were submitted to the ProteomeXchange Consortium (https://proteomecentral.proteomexchange.org/) and are available in the PRIDE partner repository with identifiers PXD059756 (*in vitro*), PXD059761 (*in vivo* plasma) and PXD059773 (*in vivo* tissue).
