# Supplementary material for: Selectivity for TP53 signalling drives the mode of action of a highly potent N,O,O-tridentate naphthoquinone-based organo-ruthenium anticancer drug candidate
Source: Chem Sci. 2025 Jul 14;16(34):15652–65. doi: 10.1039/d5sc00735f (PMC12320299; doi:10.1039/d5sc00735f)
Supplement: SC-016-D5SC00735F-s001 [file SC-016-D5SC00735F-s001.pdf]

Supplementary Information (SI) for Chemical Science.
This journal is © The Royal Society of Chemistry 2025

Supporting Information to

Selectivity for TP53 signalling drives the mode of action of a highly potent *N,O,O*-tridentate naphthoquinone-based organoruthenium anticancer drug candidate

Alexander Rosner,[1,2,‡] Lukas Skos,[2,3,‡] Theresa Mendrina,[1,4] Dina Baier,[1,4,5] Michaela Hejl,[1,3] Yasmin Borutzki,[1,2,3] Mathias Gradl,[4] Heiko Geisler,[1] Thomas Mohr,[4,6] Anton Legin,[1] Michael Jakupec,[1,5] Andrea Bileck,[3,6] Christopher Gerner,[3,6] Gunda Koellensperger,[3] Petra Heffeter,[4,5] Walter Berger,[4,5] Bernhard K. Keppler,[1,5] Wolfgang Kandioller,[1,5,] Samuel M. Meier-Menches,[1,3,6,*]*

[1] Institute of Inorganic Chemistry, University of Vienna, Waehringer Str. 42, 1090 Vienna, Austria.

[2] Doctoral School of Chemistry, University of Vienna, Waehringer Str. 38, 1090 Vienna, Austria.

[3] Department of Analytical Chemistry, University of Vienna, Waehringer Str. 38, 1090 Vienna, Austria.

[4] Centre for Cancer Research and Comprehensive Cancer Centre, Medical University Vienna, Borschkegasse 8a, Vienna, 1090, Austria.

[5] Research Cluster "Translational Cancer Therapy Research", Vienna, 1090, Austria.

[6] Joint Metabolome Facility, University of Vienna and Medical University of Vienna, Waehringer Str. 38, 1090 Vienna, Austria.

Table of contents

Materials and methods..3
Ligand synthesis..4
Complex synthesis..5
Stability and Reactivity Studies by Mass Spectrometry..10
Cell cultures..11
Viability assays..12
Cell cycle..13
Proteome profiling of compound-treated SW480 cancer cells..14
Immunoblotting..15
Immunofluorescence..16
Peptide Local Stability Assay..17
In vivo Experiments..18
nLC-MS/MS Analysis..19
Data processing and analysis..20
ICP-MS Analysis..21
Supplemental ^{1}H- and ^{13}C-NMR spectra for compound characterization..22
Supplemental ESI-MS spectra for compound characterization..27
Supplemental Figures..31
Supplementary Tables..45
References..48

Materials and methods

Chemicals

2-Hydroxy-3-(morpholinomethyl)naphthalene-1,4-dione[1] (**b**), ruthenium dimer $[RuCl_2(p\text{-cym})]_2$ and osmium dimer $[OsCl_2(p\text{-cym})]_2$ (both dimers ref.[2]) were synthesized in accordance to published procedures. Following chemicals, materials and solvents were used without further purification: ruthenium(III)chloride hydrate (Johnson Matthey), osmium tetroxide (Johnson Matthey), 1*H*-pyrazole (Acros), triethylamine (Fisher/Acros), 25% aqueous ammonium hydroxide solution (Loba), morpholine (Alfa Aesar), 37-41% aqueous formaldehyde solution (Fluka), 2-hydroxy-1,4-naphthoquinone (Acros-Fisher), acetaldehyde (Sigma-Aldrich), L-proline (Merck) and Hantzsch ester (TCI). Methanol (Sigma-Aldrich), ethyl acetate (Riedel-de Haën), ethanol (96%, Brenntag), dichloromethane (DCM) (Sigma-Aldrich) and *n*-hexane (Sigma-Aldrich) were used without further purification. DCM was dried over anhydrous calcium chloride, filtrated and stored over molecular sieve (4 Å) under inert conditions.

Instrumentation

Microwave syntheses were conducted using a Biotage® Initiator+ instrument. Compounds were purified using a Biotage® Isolera™ System with silica packed columns (silica 60, 40–63 µM, Macherey-Nagel). NMR spectra were measured on an AV III HD 700 Bruker BioSpin 700 MHz instrument or an AV III 600 Bruker BioSpin 600 MHz spectrometer. High resolution ESI mass spectra of the metalacycles were recorded at the Mass Spectrometry Center of the University of Vienna (Faculty of Chemistry) on a Bruker maXis ESI-Qq-TOF mass spectrometer. Elemental analyses were performed by the Microanalytical Laboratory of the University of Vienna with a Eurovector EA 3000(2009) equipped with a high temperature pyrolysis furnace (HT, Hekatech, Germany, 2009). Elemental analyses samples were weighed on a Sartorius SEC 2 ultra-micro balance with ±0.1 µg resolution. Sample weights of 1–3 mg were used. For calibration two NIST-certified reference materials were used: sulfanilamide ($C_6H_8N_2O_2S$) and BBOT (2, 5-bis-(5-tert-butyl-2-benzoxazol-2-yl)-thiophenone, $C_{26}H_{26}N_2O_2S$).

The limit of quantification (LOQ) was 0.05 w-% for C, H, N and 0.02 w-% for S. The presented values are the average of determinations in triplicate. UV-Vis spectra were recorded on a Perkin Elmer lambda 35 photometer, PTP (Peltier Temperature Programmer), equipped with a Julabo AWC 100 recirculating cooler.

Ligand synthesis

2-Ethyl-3-hydroxynapthalene-1,4-dione (a)

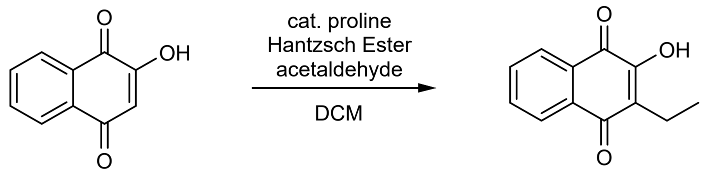

Synthesis of ligand (**a**).

Lawsone (501 mg, 2.88 mmol, 1 equiv.), Hantzsch ester (824 mg, 3.25 mmol, 1.1 equiv.) and acetaldehyde (321 µL, 253 mg, 5.74 mmol, 2 equiv.) were dissolved in dry DCM (20 mL) and stirred for five minutes at r.t.. L-Proline (66 mg, 0.57 mmol, 0.2 equiv.) was added and the yellow suspension was stirred under microwave irradiation (85 °C, 25 min). The resulting dark red organic solution was washed with 1:10 diluted hydrochloric acid and brine. The organic layer was dried over anhydrous sodium sulfate and solvent was removed under reduced pressure. Pure product was obtained *via* column chromatography (silica, gradient 40–80% DCM in *n*-hexane) as a yellow solid (463 mg, 2.29 mmol, 80%).

^{1}H NMR (600 MHz, $CDCl_3$) δ 8.13 (dd, *J* = 7.7, 1.0 Hz, 1H, CH arom. 6/9), 8.08 (dd, *J* = 7.6, 1.1 Hz, 1H, CH arom. 6/9), 7.75 (ddd, *J* = 7.6, 1.3 Hz, 1H, CH arom. 7/8), 7.68 (ddd, 1H, CH arom. 7/8), 7.27 (s, 1H, OH), 2.63 (q, *J* = 7.5 Hz, 2H, CH_2), 1.15 (t, *J* = 7.5 Hz, 3H, CH_3) ppm.

^{13}C NMR (151 MHz, $CDCl_3$) δ 184.7 (4), 181.7 (11), 152.9 (12), 135.0 (7/8), 133.1 (5/10), 133.0 (7/8), 129.6 (5/10), 126.9 (6/9), 126.2 (6/9), 126.1 (3), 16.9 (2), 12.8 (1) ppm.

Complex synthesis

General procedure

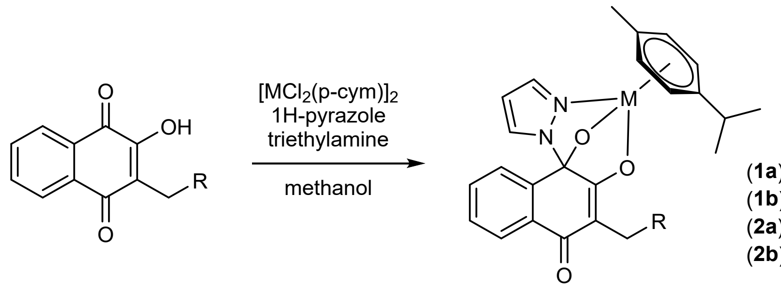

(**1a**) M= Ru, R= CH_3
(**1b**) M= Ru, R= C_4H_8NO
(**2a**) M= Os, R= CH_3
(**2b**) M= Os, R= C_4H_8NO

General procedure for synthesis of complexes (**1a**, **2a**, **1b**, **2b**).

The metal dimer (1 equiv.), pyrazole (1.9 equiv.) and the respective naphthoquinone derivative (1.9 equiv.) were dissolved in methanol (12 mL) and triethylamine (6 equiv.) was added. Stirring under microwave irradiation (60 °C, 20 min) was followed by solvent removal under reduced pressure. The crude product was purified *via* column chromatography (silica, isocratic, 70% ethyl acetate, 5% triethylamine in *n*-hexane or 10% methanol, 2% ammonium hydroxide in ethyl acetate). The product fractions were combined and the solvent was removed under reduced pressure. The formed greenish or brown hygroscopic solids were dried at 60 °C *in vacuo*.

[3-Ethyl-4-oxo-(1H-κN²-pyrazol-1-yl)-1,4-dihydronaphtalene1,2-bis(olato)-κO¹-κO²)(η⁶-p-cymene)ruthenium(II)] (1a)

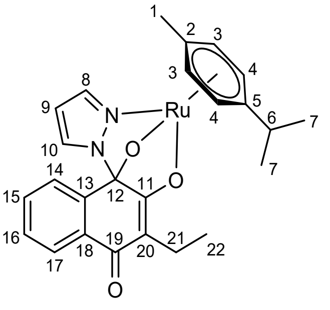

Complex **1a**.

The product was synthesized according to the general procedure, using ruthenium(II) dimer (302 mg, 0.493 mmol, 1 equiv.), 2-ethyl-3-hydroxynaphthalene-1,4-dione **a** (191 mg, 0.944 mmol, 1.9 equiv.), pyrazole (62 mg, 0.906 mmol, 1.8 equiv.) and triethylamine (410 µL, 297 mg, 2.939 mmol, 6 equiv.) in methanol (12 mL). Crude product was purified *via* column chromatography (silica, isocratic 85% ethyl acetate and 5% triethylamine in *n*-hexane). The desired compound was obtained as greenish solid (297 mg, 0.59 mmol, 74%).

^{1}H NMR (700 MHz, MeOD) δ 8.33 (d, 1H, 8), 8.14 – 8.05 (m, 1H, 14), 7.63 – 7.55 (m, 3H, 15–17), 6.68 (dd, J = 2.5, 0.4 Hz, 1H, 10), 6.34 (d, J = 2.4, 2.4 Hz, 1H, 9), 5.97 (d, J = 6.3 Hz, 1H, 4), 5.86 (d, J = 5.5 Hz, 1H, 4), 5.61 (d, J = 3.6 Hz, 2H, 3), 2.87 (hept, J = 6.9 Hz, 1H, 6), 2.41 – 2.34 (m, 1H, 21), 2.33 (s, 3H, 1), 2.32 – 2.26 (m, 1H, 21), 1.34 & 1.33 (s, 3H, 7), 0.88 (t, J = 7.4 Hz, 3H, 22) ppm.

^{13}C NMR (176 MHz, MeOD) δ 183.5 (11 or 19), 183.1 (11 or 19), 141.4 (8), 137.7 (13 or 18), 134.4 (13 or 18), 132.2 (15–17), 131.2 (15–17), 127.9 (10), 127.7 (15–17), 127.1 (14), 112.3 (20), 108.7 (9), 101.3 (5), 98.6 (2), 94.9 (12), 83.3 (4), 82.86 (4), 80.2 (3), 80.1 (3), 32.7 (6), 23.1 (7), 22.8 (7), 18.3 (1), 16.9 (21), 13.7 (22) ppm.

ESI-HR-MS [M+H^{+}] m/z found: 505.1059 (505.1065), [M+Na^{+}] m/z found: 527.0884 (527.0879).

Elemental analysis calculated for $C_{25}H_{26}N_2O_3Ru \cdot 0.35\ H_2O$: C 58.89%, H 5.28%, N 5.49%, O 10.51%;

Found elemental composition: C 58.55%, H 5.26%, N 5.57%, O 10.25%.

[3-(Morpholinomethyl)-4-oxo-(1H-κN^2-pyrazol-1-yl)-1,4-dihydronaphtalene-1,2-bis(olato)-κO^1-κO^2)(η^6-p-cymene)ruthenium(II)] (1b)

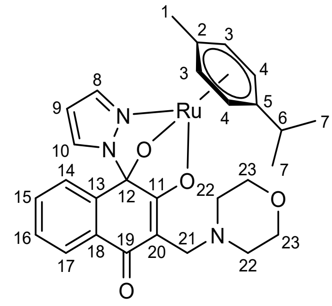

Complex **1b**.

The product was synthesized according to the general procedure, using ruthenium(II) dimer (308 mg, 0.503 mmol, 1 equiv.), 2-hydroxy-3-(morpholinomethyl)naphthalene-1,4-dione **b** (308 mg, 0.967 mmol, 1.9 equiv.), pyrazole (68 mg, 1 mmol, 2 equiv.) and triethylamine (10 µL, 297 mg, 2.939 mmol, 6 equiv.) in methanol (12 mL). Crude product was purified *via* column chromatography (silica, isocratic 10% methanol and 2% ammonium hydroxide in ethyl acetate). The desired compound was obtained as a brownish oil, which solidified during drying *in vacuo* at 60 °C (295 mg, 0.51 mmol, 51%).

^{1}H NMR (600 MHz, MeOD) δ 8.35 (d, J = 2.1 Hz, 1H, 8), 8.14 – 8.09 (m, 1H, 14), 7.67 – 7.61 (m, 3H, 15–17), 6.73 – 6.71 (m, 1H, 10), 6.36 – 6.34 (m, 1H), 9), 5.99 (d, J = 5.9 Hz, 1H, 4), 5.91 (d, J = 6.7 Hz, 1H, 4), 5.64 (d, J = 5.9 Hz, 1H, 3), 5.58 (d, J = 5.9 Hz, 1H, 3), 3.58 – 3.52 (m, 4H, 23), 3.50 (d, *J* = 12.4 Hz, 1H, 21), 3.41 (d, *J* = 12.4 Hz, 1H, 21), 2.86 (hept, J = 6.9 Hz, 1H, 6), 2.45 – 2.35 (m, 2H, 22), 2.33 (s, 3H, 1), 2.27 – 2.20 (m, 2H, 22), 1.34 & 1.33 (d, J = 2.1 Hz, 3H, 7) ppm.

^{13}C NMR (150.95 MHz, MeOD) δ 186.3 (11 or 19), 183.7 (11 or 19), 141.6 (8), 137.7 (13 or 18), 134.0 (13 or 18), 132.6 (15-17), 131.4 (15-17), 128.1 (10), 127.8 (15-17), 127.4 (14), 109.0 (9), 104.3 (20), 100.8 (5), 98.8 (2), 95.0 (12), 83.8 (4), 83.1 (4), 80.1 (3), 78.0 (3), 67.4 (23), 54.0 (22), 50.6 (21), 32.6 (6), 23.2 (7), 22.8 (7), 18.5 (1) ppm.

ESI-HR-MS [M+H^+] m/z found: 576.1427 (576.1436).

Elemental analysis calculated for $C_{28}H_{31}N_3O_4Ru \cdot 0.85\ H_2O$: C 57.01%, H 5.59%, N 7.12%, O 13.15%;

Found elemental composition: C 56.73%, H 5.33%, N 7.28%, O 12,94%.

[3-Ethyl-4-oxo-(1H-κN^2-pyrazol-1-yl)-1,4-dihydronaphtalene1,2-bis(olato)-κO^1-κO^2)(η^6-p-cymene)osmium(II)] (2a)

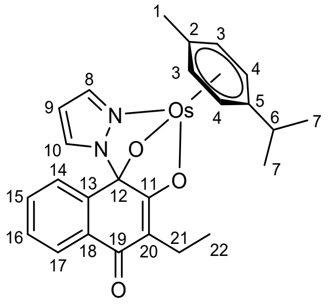

Complex **2a**.

The product was synthesized according to the general procedure, using osmium(II) dimer (200 mg, 0.253 mmol, 1 equiv.), 2-ethyl-3-hydroxynaphthalene-1,4-dione **a** (99 mg, 0.488 mmol, 1.9 equiv.), pyrazole (33 mg, 0.477 mmol, 1.9 equiv.) and triethylamine (212 µL, 154 mg, 1.518 mmol, 6 equiv.) in methanol (12 mL). Crude product was purified *via* column chromatography (silica, isocratic 85% ethyl acetate and 5% triethylamine in *n*-hexane). Desired compound was obtained as a greenish solid (219 mg, 0.37 mmol, 73%).

^{1}H NMR (600 MHz, MeOD) δ 8.29 (d, 1H, 8), 8.16 – 8.11 (m, 1H, 14), 7.66 – 7.59 (m, 3H, 15–17), 6.87 (dd, J = 2.5, 0.4 Hz, 1H, 10), 6.41 – 6.37 (m, 1H, 9), 6.18 (d, J = 5.4 Hz, 1H, 4), 6.09 (d, J = 5.5 Hz, 1H, 4), 5.84 (d, J = 5.5 Hz, 1H, 3), 5.81 (d, J = 5.5 Hz, 1H, 3), 2.74 (hept, 1H, 6), 2.40 (s, 3H, 1), 2.39 – 2.28 (m, 2H, 21), 1.32 & 1.30 (d, J = 1.6 Hz, 3H, 7), 0.88 (t, J = 7.4 Hz, 3H, 22) ppm.

^{13}C NMR (151 MHz, MeOD) δ 183.8 (11 or 19), 183.0 (11 or 19), 141.4 (8), 136.7 (13 or 18), 134.5 (13 or 18), 132.4 (15–17), 131.6 (15–17), 127.8 (15–17), 127.6 (10), 127.3 (14), 112.0

(20), 109.4 (9), 98.2 (12), 91.1 (5), 89.0 (2), 73.8 (4), 73.2 (4), 70.4 (3), 70.3 (3), 33.1 (6), 23.5 (7), 23.1 (7), 18.6 (1), 17.0 (21), 13.6 (22) ppm.

ESI-HR-MS [M+H$^+$] m/z found: 595.1620 (595,1628), [M+Na$^+$] m/z found: 617.1440 (617.1447).

Elemental analysis calculated for $C_{25}H_{26}N_2O_3Os \cdot 0.3\ H_2O$: C 50.20%, H 4.48%, N 4.68%, O 8.83%;

Found elemental composition: C 49.93%, H 4,38%, N 4.68%, O 8.61%.

[3-(Morpholinomethyl)-4-oxo-(1H-κN²-pyrazol-1-yl)-1,4-dihydronaphtalene-1,2-bis(olato)-κO¹-κO²)(η⁶-p-cymene)osmium(II)] (**2b**)

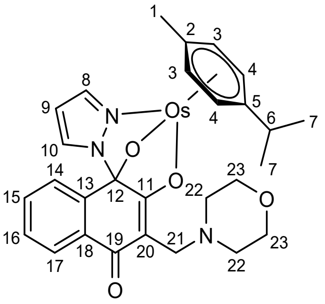

Complex **2b**.

The product was synthesized according to the general procedure, using osmium(II) dimer (202 mg, 0,255 mmol, 1 equiv.), 2-hydroxy-3-(morpholinomethyl)naphthalene-1,4-dione **b** (136 mg, 0.498 mg, 1.9 equiv.), pyrazole (33 mg, 0,485 mmol, 1.9 equiv.) and triethylamine (212 µL, 154 mg, 1.518 mmol, 6 equiv.) in methanol (12 mL). Crude product was purified *via* column chromatography (silica, isocratic 10% methanol and 2% ammonium hydroxide in ethyl acetate). Desired compound was obtained as a brownish oil, which solidified during drying *in vacuo* at 60 °C (178 mg, 0.51 mmol, 51%).

^{1}H NMR (600 MHz, MeOD) δ 8.31 (d, J = 2.2 Hz, 1H, 8), 8.18 – 8.14 (m, 1H, 14), 7.70 – 7.64 (m, 3H, 15–17), 6.92 (d, J = 2.6 Hz, 1H, 10), 6.42 – 6.40 (m, 1H, 9), 6.21 (d, J = 5.5 Hz, 1H,

4), 6.14 (d, J = 5.5 Hz, 1H, 4), 5.86 (d, J = 6.3 Hz, 1H, 3), 5.79 (d, J = 5.5 Hz, 1H, 3), 3.57 – 3.53 (m, 3H, 23), 3.52 – 3.40 (m, 2H, 21), 2.74 (hept, J = 6.9 Hz, 1H, 6), 2.42 – 2.22 (m, 4H, 22), 2.40 (s, 3H, 1) 1.32 & 1.31 (d, J = 1.6 Hz, 3H, 7) ppm.

^{13}C NMR (150.95 MHz, MeOD) δ 186.0 (11 or 19), 184.3 (11 or 19), 141.7 (8), 136.6 (13 or 18), 134.1 (13 or 18), 132.8 (15–17), 131.6 (15–17), 127.8 (15–17), 127.8 (10), 127.6 (14), 109.7 (9), 104.1 (20), 98.4 (12), 90.6 (5), 89.2 (2), 74.5 (4), 73.6 (4), 70.3 (3), 70.2 (3), 67.4 (23), 54.1 (22), 50.7 (21), 33.1 (6), 23.5 (7), 23.2 (7), 18.8 (1) ppm.

ESI-HR-MS [M+H$^+$] m/z found: 666.1994 (665.1920).

Elemental analysis calculated for $C_{28}H_{31}N_3O_4Os \cdot 1.1\ H_2O$: C 49.19%, H 4.90%, N 6.15%, O 11.94%;

Found elemental composition: C 48.87%, H 4.73%, N 6.39%, O 11.71%.

Stability Study by UV-Vis Spectroscopy

All four compounds were freshly diluted in PBS (1% DMSO) from a DMSO stock solution at 20 °C. Compounds **1a**/**2a** were diluted to 40 µM, while compounds **1b**/**2b** were diluted to 80 µM. The stability of the compounds was monitored in a UV-Vis spectrophotometer (Perkin Elmer lambda 35 photometer) over 96 h by acquiring an UV-Vis spectrum every 30 mins. The percentage of the remaining intact compound was calculated by the ratio of the maximum absorbance of the tridentate complexes' characteristic absorption band around 350–370 nm after a given timepoint (A) compared to the one directly after dilution (A_0).

Stability and Reactivity Studies by Mass Spectrometry

All four compounds were freshly prepared at 40 mM in DMSO. They were diluted in H_2O (VWR, LC-MS grade) to obtain 50 µM solutions in triplicates per compound. Thereof, one

aliquot was used for stability testing of the respective test substance in aqueous solution. Another aliquot was spiked with an equimolar ubiquitin solution (50 µM in H_2O) to test the interaction with this protein. Further, an aliquot of each of the four compounds **1a**, **1b**, **2a** and **2b** were equimolarly added (50 µM) to a mixture of three amino acids and two nucleotides (Cys/Met/His/ATP/GTP; each 50 µM in aqueous solution) to test the interaction with small biological nucleophiles. All samples were mixed and incubated in a shaking incubator at 37 °C with speed set to 1000 rpm in the dark (Eppendorf ThermoMixer® C). After 1.5 h, 24 h and 72 h, 300 µL per sample were transferred to fresh reaction vessels, immediately frozen in liquid N_2 and stored at –20 °C until measurement.

Samples were diluted to a concentration of 2.5 µM with an organic eluent (49.5% v/v acetonitrile, 49.5% v/v methanol,1% v/v H_2O), except ubiquitin containing samples which were diluted to the same concentration with acidified H_2O (0.1% v/v formic acid). The samples were then analysed by flow-injection using an amazon speed ETD ion trap (Bruker Daltonics) in the positive ion mode over 0.4 min using the following experimental parameters: HV capillary ±4.5 kV, end plate offset –500 V, dry temp 180 °C, nebulizer 3 bar, dry gas 5 L min^{-1}, scan range *m/z* 200–1400, target mass *m/z* 900, flow rate 5 µL min^{-1}. For ubiquitin containing samples, the scan range was adapted to *m/z* 300–2200 with a target mass of *m/z* 1100. Data was acquired using trapControl (Version 7.1.83.0) and processed using DataAnalysis (Version 4.1.359.0).

Cell cultures

SW480 human colon carcinoma cells were obtained from the American Type Culture Collection (ATCC, Manassas, VA, USA) and cultured in Eagle's minimum essential medium (Sigma Aldrich), supplemented with heat-inactivated fetal bovine serum (10% v/v, Gibco), MEM non-essential amino acid solution 100× (1% v/v, Gibco), sodium pyruvate 100 mM (1% v/v, Gibco), and penicillin−streptomycin solution 100× (1% v/v, Gibco, containing penicillin G sodium salt at 10'000 units mL^{-1} and streptomycin sulphate at 10 mg mL^{-1}). The cells were

cultured in adherent T75 flasks (Sarstedt) in a humidified incubator at 37 °C and 5% CO_2 (HeraCell150i, Thermo Fisher Scientific) and subcultured three times a week as the cells reached 75–80% growth area occupied.

HCT116 human colon carcinoma cells (from ATCC) as well as a p53KO variant thereof (kindly provided by B. Vogelstein, Ludwig Center at Johns Hopkins University, Baltimore, MD, USA) were grown in McCoy's 5A medium (Sigma-Aldrich) supplemented with 4 mM L-glutamine (Sigma-Aldrich) and 10% heat-inactivated foetal bovine serum (Serana, Pessin, Germany). CT-26 murine colon carcinoma cells (from ATCC) were grown in Dulbecco's modified minimum essential medium/Ham's F12 nutrient mix (DMEM/F12, Sigma-Aldrich) supplemented with 4 mM L -glutamine (Sigma-Aldrich) and 10% heat-inactivated foetal bovine serum (Serana). These cell lines were cultured in adherent T75 flasks (Starlab, Hamburg, Germany) in a humidified incubator (Binder CB210) at 37 °C and 5% CO_2 and subcultured twice a week. Trypsin/EDTA solution (0.25%, Sigma-Aldrich) was used for detachment of all cell lines.

Viability assays

SW480 human colon carcinoma cells (4.000 cells/well for the ethyl complexes; 4.000 and 10.000 cells/well for the morpholino complexes) were seeded in flat-bottom 96-well plates (Corning) and incubated at 37 °C for 24 h in a humidified incubator at 5% CO_2 (HeraCell150i, Thermo Fisher Scientific). Stock solutions of the compounds were freshly prepared in DMSO (10 mM **1a**; 10 mM **1b**; 40 mM **2a**; 40 mM **2b**) and diluted to 0.01, 0.05, 0.1, 0.2, 0.5, 1, 5 and 10 µM in complete cell culture medium for the ethyl compounds and 5, 10, 20, 40, 60, 80, 120, 160 µM for the morpholino compounds (final concentration of DMSO ≤0.4% v/v). The cells were incubated with the compounds for 24 h and 72 h. Then, the cell metabolism was colorimetrically determined using 3-(4,5-dimethylthiazol-2-yl)-2,5-diphenyl tetrazolium bromide (MTT). For this purpose, MTT reagent (20 µL, 5 mg mL^{-1} in phosphate-buffered saline, PBS) was added to each well and further incubated for 4 h. The medium was gently

removed and 100 µL DMSO was added to dissolve the formazan crystals. Photometric quantification was performed at 570 nm with a Multiskan Go 1510 photometer (Thermo Fisher Scientific).

The absorbances were blank corrected and converted to relative proliferation. The IC_{50} values (concentration of 50% growth inhibition) were obtained using a sigmoidal fit of the experimental data and calculated as the mean ± standard deviation of at least three independent experiments, each in at least triplicates.

HCT116, HCT116 p53KO (each 2.000 cells/well) and CT-26 cells (500 cells/well) were seeded in 100 µL/well into flat-bottom 96-well plates (Starlab) and incubated at 37 °C for 96 h in a humidified incubator (Binder CB210) at 5% CO_2. The test compound was dissolved in DMSO, serially diluted in the appropriate cell culture medium (see section above) to final DMSO concentrations of ≤0.5% v/v and 100 µL aliquots added to wells in triplicates. After incubation for 96 h, the drug-containing media were replaced with 100 µL/well of a 1 : 6 MTT (in PBS) / RPMI 1640 mixture and plates incubated for 4 h. Then, the supernatants were removed and formazan crystals dissolved in 150 µL of DMSO per well. Optical densities were measured with a microplate reader (ELx808, Biotek) at a wavelength of 550 nm (and a reference wavelength of 690 nm) and blank corrected. The IC_{50} values were interpolated from concentration-effect curves and averaged from at least three independent experiments.

For combination drug testing using **1a** and bafilomycin or bortezomib, SW480 or CT26 cells (each 3.000 cells/well) were seeded in 100 µL/well into flat-bottom 96-well plates (Starlab) and incubated at 37 °C for 24 h in a humidified incubator (Binder CB210) at 5% CO_2. Complex **1a**, bafilomycin or bortezomib were dissolved in DMSO and diluted in appropriate cell culture medium to respective concentrations. After 24 h of treatment, plates were measured by MTT assay as described above.

Cell cycle

Colon carcinoma cells (SW480) were seeded into 12-well plates (CytoOne, Starlab) in densities of 1×10^5 cells per well (in 1 mL complete medium). After 24 h, cells were treated with

different concentrations of **1a**, which was freshly dissolved in DMSO and instantly diluted in medium. Etoposide (causing G2/M phase arrest) and gemcitabine (causing G1/S arrest) were applied as positive controls. Plates were incubated at 37 °C, 5% CO_2 in a moist atmosphere for 24 h. Following the exposure, cells were collected via *trypsinization*, washed with supplemented medium and PBS, and subsequently stained with the DNA-intercalating dye propidium iodide (PI) diluted in hypotonic fluorochrome solution (HFS: 0.1% v/v Triton X-100, 0.1% w/v sodium citrate in H_2O; PI/HFS mixture: 40 µg mL^{-1}, 500 µL per probe). The PI solution was added to the HFS shortly before use and the cells were stained in the dark at 4 °C overnight. The fluorescence of all samples was measured no longer than 20 h after staining by means of flow cytometry (Guava easyCyte 8HT, Millipore).

Proteome profiling of compound-treated SW480 cancer cells

SW480 human colon carcinoma cells were seeded in adherent T25 flasks (Sarstedt) at 2×10^6 cells per well and left to adhere in complete cell culture medium. After 24 h, the medium was exchanged with fresh complete medium (DMSO mock control) or fresh medium containing freshly dissolved compound (0.17 µM **1a**, 0.26 µM **2a**, 34 µM **1b**, 43 µM **2b** from 40 mM stock solution in DMSO, respectively) and incubated for another 24 h. Each condition was carried out in hexuplicates. The cells were processed according to a nucleocytoplasmic fractionation protocol as previously described.[3] All steps were performed on ice. The medium was removed, and the cells were washed twice with 1×PBS (phosphate buffered saline, 3 mL). Then, PBS was thoroughly removed. Isotonic lysis buffer (10 mM HEPES, 10 mM NaCl, 3.5 mM $MgCl_2$, 1 mM EGTA, 0.25 M Sucrose, 0.5% Triton X-100) containing protease inhibitors (1% PMSF, Sigma, and 1% protease and phosphatase inhibitor cocktail, Roche) was added to the flasks, the cells were scraped off and transferred into labelled 15 mL Falcon tubes. The cellular membrane was ruptured using shear stress by pressing the cell suspension through a syringe 9–12 times. Membrane rupture with intact nuclei was monitored under a microscope. After centrifugation (960 g, 5 min), the supernatant containing the cytoplasmic (CYT) proteins was transferred into new tubes containing ice-cold ethanol (1 : 5, HPLC grade) and precipitated

overnight at –20 °C. The pellet containing the nuclei was incubated with a hypertonic solution (10 mM Tris-HCl, 1 mM EDTA and 0.5 M NaCl) and subsequently diluted 1:10 with NP-40 buffer (10 mm Tris-HCl, 1 mm EDTA and 0.5% Triton X-100) containing protease inhibitors (1% PMSF, Sigma, and 1% protease and phosphatase inhibitor cocktail, Roche). After centrifugation (960 g, 5 min), the soluble nuclear (NE) proteins were also transferred into new tubes containing ice-cold ethanol (1 : 5, HPLC grade) and precipitated overnight at –20 °C. The precipitated proteins were finally pelleted by centrifugation (5000 g, 30 min, 4 °C), the solution was decanted and the pellet dried under vacuum.
Samples were then dissolved in lysis buffer (8 M urea, 20% SDS, 1 M TEAB at pH 7.55) and denatured at 95 °C for 5 min. The protein amount was then quantified using a BCA assay. A total of 20 µg protein per sample was used for processing. Proteins were reduced with 50 µL of DTT (64 mM) and carbamidomethylated with 50 µL of iodoacetamide (486 mM). Proteolytic digestion was performed using the ProtiFi S-trap technology.[4] Briefly, samples were loaded onto S-trap mini cartridges followed by the addition of trapping buffer (methanol 90% v/v, 0.1 M TEAB). Afterwards. samples were thoroughly washed and digested with 0.5 µg trypsin/Lys-C mix (Promega) at 37 °C for 2 h (1 : 40 enzyme-to-protein ratio). Peptides were eluted, dried in a SpeedVac and stored at –20 °C.

Immunoblotting

SW480 cells (2×10^6) were seeded in 1 mL medium in 6-well plates in a total volume of 2 mL and allowed to adhere overnight. Cells were treated for 24 h with respective compounds at indicated concentrations. For whole cell lysates, samples were collected, proteins isolated, and Western blotting performed as described in detail previously.[5] In brief, protein concentration was determined using the Micro BCA™ Protein Assay Kit (Thermo Fisher Scientific, Waltham, MA, USA). Proteins were separated using sodium dodecyl sulfate polyacrylamide gel electrophoresis and subsequently transferred onto polyvinylidene difluoride membranes (Thermo Fisher Scientific). Primary antibodies p53 (DO-1) (1 : 200, MS-187-P0) and p21 (12D1) (1 : 800, #2947) were purchased from Thermo Fisher Scientific or

Cell Signaling Technology (Danvers, MA, USA), respectively. Horseradish-peroxidase-linked secondary antibodies, anti-mouse IgG (Fc specific) antibody (#7076S) and anti-rabbit IgG antibody (#7074S) were purchased from Cell Signaling Technology (Danvers, MA, USA). Anti-ß-actin (AC-15) (A5441, 1:2000) was obtained from Sigma-Aldrich (St. Louis, MO, USA). Protein expression was quantified using Image J (version 1.54p).

Immunofluorescence

SW480 and CT26 cells were grown at a density of 6.7×10^4 cells/mL on 8-well glass coverslips (80841, ibidi GmbH, Gräfelfing, Germany) in 300 µL complete media. The next day, cells were treated with respective compounds for 24 h. Cells were fixed with 4%(v/v) paraformaldehyde for 15 min at RT, followed by washing, blocking and permeabilization with 5%(w/v) BSA and 0.3% (w/v) Triton-X-100 for 60 min. Primary antibodies p53 (DO-1) (1 : 100) or p21 (12D1) (1 : 500) were diluted in 1% BSA-0.3% Triton-X-100 in PBS and incubated overnight at 4 °C. One well was incubated without primary antibody as background control. Cells were washed and stained with secondary goat anti-mouse AlexaFlour-488 (1 : 500 dilution, A-11029, Thermo Fisher Scientific) or goat anti-rabbit AlexaFlour-488 (1 : 500 dilution, A-11034, Thermo Fisher Scientific) antibodies for 1 h at RT. Following, cells were stained with 0.2% Wheat Germ Agglutinin (WGA)-Rhodamine (RL-1022, Vector Laboratories, Inc.) and 0.1% DAPI (62248, Thermo Fisher Scientific) for staining of plasma membrane and nuclei, respectively, according to manufacturer's recommendations. Slides were washed and covered using VECTASHIELD® HardSet™ Antifade Mounting Medium (VECH-1400, Szabo Scandic, Vienna, Austria). Images were acquired on a Zeiss LSM 800 Confocal Laser Scanning Microscope equipped with an Pln Apo 63×/1.4 Oil DICII objective using Zeiss ZEN blue software (version 3.4.91.00000, Carl Zeiss Microscopy GmbH). Laser intensities and detector gains were maintained at the same level during all imaging sessions. Signal intensity calculations and TP53/DAPI weighed co-localization coefficient analysis were done using ImageJ or ZEN blue software, respectively.

Peptide Local Stability Assay

SW480 cancer cells were cultivated in complete medium as above in two T75 adherent flasks. The protein extraction was performed on ice. The flasks were washed twice with PBS (1×, 5 mL) and the wash solution was completely removed. Then, PBS (1×, 2mL, containing 1% PPC) was added in each T75 flak. The cells were scraped off and the cell suspension was transferred to 15 mL falcon tubes. After four freeze-thaw cycles using liquid nitrogen and a water bath (37 °C), the falcons were centrifuged (3500 rpm, 5 min, 4 °C) to pellet cell debris. The clear supernatant was pooled in a 5 mL Eppendorf tube and kept on ice. The protein concentration was determined using a BCA assay as described above and adjusted to 1 µg/µL using PBS (1×, containing 1% PPC). Then, 50 µL (= 50 µg protein) were aliquoted into individual Eppendorf tubes (4x untreated, 4x **1a**-treated, 4x plecstatin-1 treated). Stock solutions of **1a** (50 µM in DMSO) and plecstatin-1 (1 mM in DMSO) were prepared. One microliter of DMSO or compound solution was added to each condition giving final concentrations of **1a** at 1 µM and plecstatin-1 at 20 µM. The 12 samples were incubated for 2 h at 4 °C under constant shaking (800 rpm). Thereafter, partial native trypsinization was performed at an enzyme-to-substrate ratio of 1 : 2.2 with trypsin/LysC (Promega) for 60 s at 37 °C and under constant shaking (1000 rpm). Then, 8M guanidine HCl (165 µL, in 60 mM HEPES, pH 8.2) was added to quench the digestion. The peptidic samples were reduced with tris-(2-carboxyethyl)-phosphine (12 µL, 200 mM) and alkylated with 2-chloroacetamide (12 µL, 800 mM). The samples were heated to 95 °C for 5 mins under constant shaking (1400 rpm). The sample solutions were then concentrated in a SpeedVac until reaching around 50 µL, diluted with 100 µL styrenedivinylbenzene-reverse phase sulfonate (SDB-RPS) loading buffer (99% iPrOH, 1% TFA) and finally desalted *via* SDB-RPS StageTips. This step also removes undigested proteins. Peptides were eluted with freshly prepared SDB-RPS elution buffer (2.4 mL ACN, 1.6 mL H_2O, 20 µL NH_4OH), completely dried in a SpeedVac and stored at –20 °C.

In vivo Experiments

Animals. All experiments were carried out with male Balb/c mice according to the regulation of the Ethics Committee for the Care and Use of Laboratory Animals at the Medical University Vienna (BMWF-2022-0.770.291), the guidelines from the Austrian Animal Science Association and the Federation of European Laboratory Animal Science Associations (FELASA). The animals were kept in a pathogen-free conditions, controlled environment with 12 h light–dark cycle with *ad libitum* access to food and water. Every procedure was done in a laminar airflow cabinet.

Treatment. CT-26 cells (5×10^5 in 50 µL serum-free medium) were injected subcutaneously (s.c.) into the right flank of ten– to eleven-week-old male Balb/c mice. Three days after cell injection, all compounds (dissolved in 10% DMSO in water) were administered intraperitoneally (i.p.) in a concentration of 10 mg/kg in case of **1a**/**2a** and 30 mg/kg in case of **1b**/**2b**. Regarding anticancer activity experiments, mice were treated on 5 consecutive days over 2 weeks. Four hours after the last treatment **1a**-treated mice were sacrificed and analysed for ruthenium levels in tissue *via* ICP-MS measurements. In case of the proteomic profiling experiment, mice were treated with **1a** in a concentration of 10 mg/kg dissolved in 10% DMSO in water for 5 consecutive days. On the last day, 2 h after the last treatment, mice were euthanized, and blood was collected in an EDTA tube via heart punctation. Tumours and livers were harvested, quickly rinsed with PBS and snap-frozen in liquid nitrogen. Blood plasma was obtained by centrifugation (2×10 min at 900 g). Samples were stored at –80 °C until processing.

Histological evaluation. Liver tissues from mice treated for 5 days (control and **1a**) were fixed in 4% formaldehyde (Carl Roth, #P087.3) after dissection, paraffin-embedded and sliced in 3.5 µm thick sections. Hematoxylin and eosin (H&E) staining was performed as previously described in Feldman and Wolfe *et al.*,[6] followed by scanning with a 3D Histech Microscopic High Throughput slide scanner for Brightfield. The Papanicolaou's solution 1a Harris'

hematoxylin solution as well as the Eosin Y disodium salt were purchased from MERCK. The Scott's solution was bought from Morphisto.

Sample Processing. All steps were performed on ice. The frozen tissue samples (weights ranging from 8.6–51.5 mg) were transferred to 15 mL Falcon tubes containing 150 µL sodium deoxycholate lysis buffer (SDC; 0.4 g SDC, 500 µL Tris·HCl (pH 8.8) in 9.5 mL H_2O) and homogenized with a sonication probe (Bandelin Sonoplus HD 2070; Bandelin Electronic GmbH & Co. KG, Berlin, Germany). After sonication the homogenates were centrifuged at 10.000 g for 5 minutes and the resulting supernatant was transferred to new reaction tubes. Tissue samples were diluted at a ratio of 1 : 10, heated at 95 °C for 5 min and stored at –20 °C. EDTA-anticoagulated plasma samples were diluted in SDC buffer at a ratio of 1 : 20, heated at 95 °C for 5 min and stored at –20 °C. The protein concentration of each sample was determined using a BCA assay. The proteolytic digestion was performed according to an in-solution protocol.[7] Twenty µg of protein was reduced and alkylated with 10 µL of a mixture containing 100 mM tris(2-carboxyethyl)phosphine (TCEP) and 400 mM 2-chloroacetamide (2-CAM) for 5 min at 45 °C, followed by 18 h digestion with Trypsin/Lys-C (1:100 enzyme-to-substrate ratio) at 37 °C. Next, samples were concentrated in a vacuum concentrator, diluted with 100 µL styrenedivinylbenzene-reverse phase sulfonate (SDB-RPS) loading buffer (99% iPrOH, 1% TFA) and then desalted *via* SDB-RPS StageTips. Peptides were eluted with freshly prepared SDB-RPS elution buffer (2.4 mL ACN, 1.6 mL H_2O, 20 µL NH_4OH), completely dried in a SpeedVac and then stored at –20 °C.

nLC-MS/MS Analysis

Nano-LC-MS/MS analysis was performed as described previously.[8] Briefly, dried samples in glass inserts were dissolved with 5 µL of a peptide standard mix consisting of four synthetic peptides at 10 fmol in 30% formic acid (Glu1-fribrinopeptideB, EGVNDNEEGFFSAR; M28, TTPAVLDSDGSYFLYSK; HK0, VLETKSLYVR and HK1, VLETK(ε-Ac)SLYVR) and diluted

with loading buffer (40 µL, acetonitrile 2% v/v, TFA 0.05% v/v; ddH_2O 97.95% v/v). The glass inserts were transferred into 2 mL Eppendorf tubes filled with 400 µL ddH_2O, sonicated for 5 min (20% power), centrifuged (5000 g, 7 min, RT) and transferred into labelled HPLC vials. MS data was acquired employing a timsTOF Pro mass spectrometer (Bruker Daltonics, Bremen, Germany) hyphenated to a Dionex UltiMate™ 3000 RSLCnano system (Thermo Scientific, Bremen, Germany). Samples were analysed using data-dependent acquisition and label free quantification (LFQ) shotgun proteomics in PASEF mode.[9] From each sample 5 µL were loaded on an Acclaim™PepMap™ C18 HPLC pre-column (2 cm × 100 µm, 100 Å, Thermo Fisher Scientific™, Vienna, Austria) at a flow rate of 10 µL min^{-1}. After trapping, peptides were eluted at a flow rate of 300 nL min^{-1} and chromatographic separation was achieved on an Aurora series CSI UHPLC emitter column (25 cm × 75 µm, 1.6 µm C18, Ionopticks, Fitzroy, Australia) applying a gradient from 7–40% of mobile phase B (79.9% ACN, 20% H2O, 0.1% FA) in mobile phase A (99.9% H2O, 0.1% FA). For *in vitro* samples and *in vivo* tissue proteomics, a gradient over 90 min was used giving a total run time of 135 min per sample. In vivo plasma samples were analysed using a 43 min gradient giving a total run time of 85 min. Additionally, *in vivo* samples were measured in technical duplicates.

Data processing and analysis

Raw data obtained from the timsTOF Pro MS was processed based on LFQ proteomics by MaxQuant (Version 1.6.17.0),[10] including the built-in Andromeda search engine, and searched against the UniProt Database.[11] Separate searches for the *in vitro* and *in vivo* data sets were conducted by using a fasta-file for the human proteome (version 11/2021, 20'375 entries) and a fasta-file for the mouse proteome (version 06/2021, 17'519 entries), respectively. Only non-redundant Swissprot entries with at least two identified peptides per protein were used for identifying protein groups. The first and main search peptide tolerance was 50 and 25 ppm, respectively. The false discovery rate (FDR) was fixed to 0.01 on the peptide and protein level. Match between runs was enabled with an alignment time window of 0.7 min. Oxidation of methionine and N-terminal acetylation were set as variable modifications whereas

carbamidomethylation of cysteines was set as fixed modification. The statistical evaluation was performed with Perseus (Version 1.6.14)[12] using LFQ intensities of the MaxQuant result file. After filtering potential contaminants the LFQ values were Log(2)-transformed. Prior to Log(2)-transformation, the mean of the *in vivo* technical duplicates was calculated. Only those protein groups were considered for data evaluation, which were detected at least five times in at least one condition. Missing data points were imputed. Permutation-based FDR was set to 0.05 and S0 = 0.1 for multi-parameter corrected significance testing of protein regulation. The final dataset was further analysed using the web-based applications DAVID bioinformatics Resources (Version 6.8)[13] and STRING (Version 10.0).[14]

Additionally, statistical analysis of protein regulations from the *in vivo* samples was performed using the LIMMA R-package.[15] P-values where adjusted according to Benjamini-Hochberg[16] and a gene set variation analysis was performed (GSVA).[17] Protein Sets where constructed using the molecular signature database.[18]

ICP-MS Analysis

Total metal content in organs harvested from **1a**-treated mice was determined by inductively coupled plasma mass spectrometry (ICP-MS) on an Agilent 7800 instrument and Agilent SPS 4 autosampler using the following parameters: RF power 1550, carrier gas (Ar) 1.08 $L \cdot min^{-1}$, plasma gas (Ar) 15 $L \cdot min^{-1}$, dwell time 0.1 s, 12 replicates with 100 sweeps. A nickel cone was used and ^{101}Ru, ^{115}In and ^{185}Re isotopes were monitored. Data was processed by Agilent MassHunter (Version C.01.04) software.

All ^{101}Ru-values were blank corrected. The lower limit of quantification of the method was 0.071 µg L^{-1}. Organs, tumour and blood were digested with nitric acid (20% w/w) and diluted with ultrapure water to reach a final nitric acid concentration <4%.

Supplemental ^{1}H- and ^{13}C-NMR spectra for compound characterization

2-Ethyl-3-hydroxynapthalene-1,4-dione (**a**)

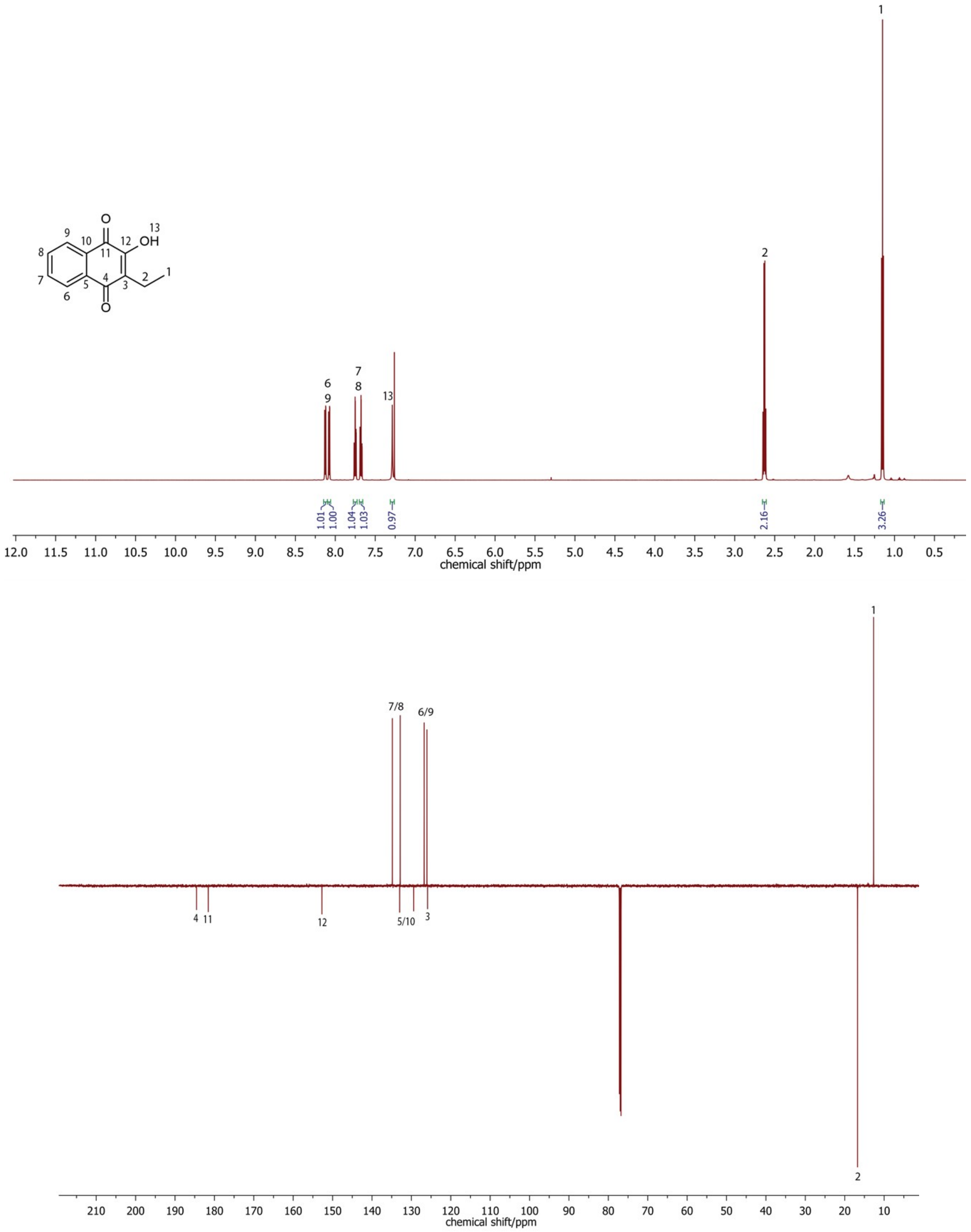

Suppl. Figure S1. Structure and atom numbering (above), ^{1}H-NMR (centre) and ^{13}C-NMR (below) in $CDCl_3$.

[3-Ethyl-4-oxo-(1H-κN^2 -pyrazol-1-yl)-1,4-dihydronaphtalene1,2-bis(olato)-κO^1-κO^2)(η^6-p-cymene)ruthenium(II)] (**1a**)

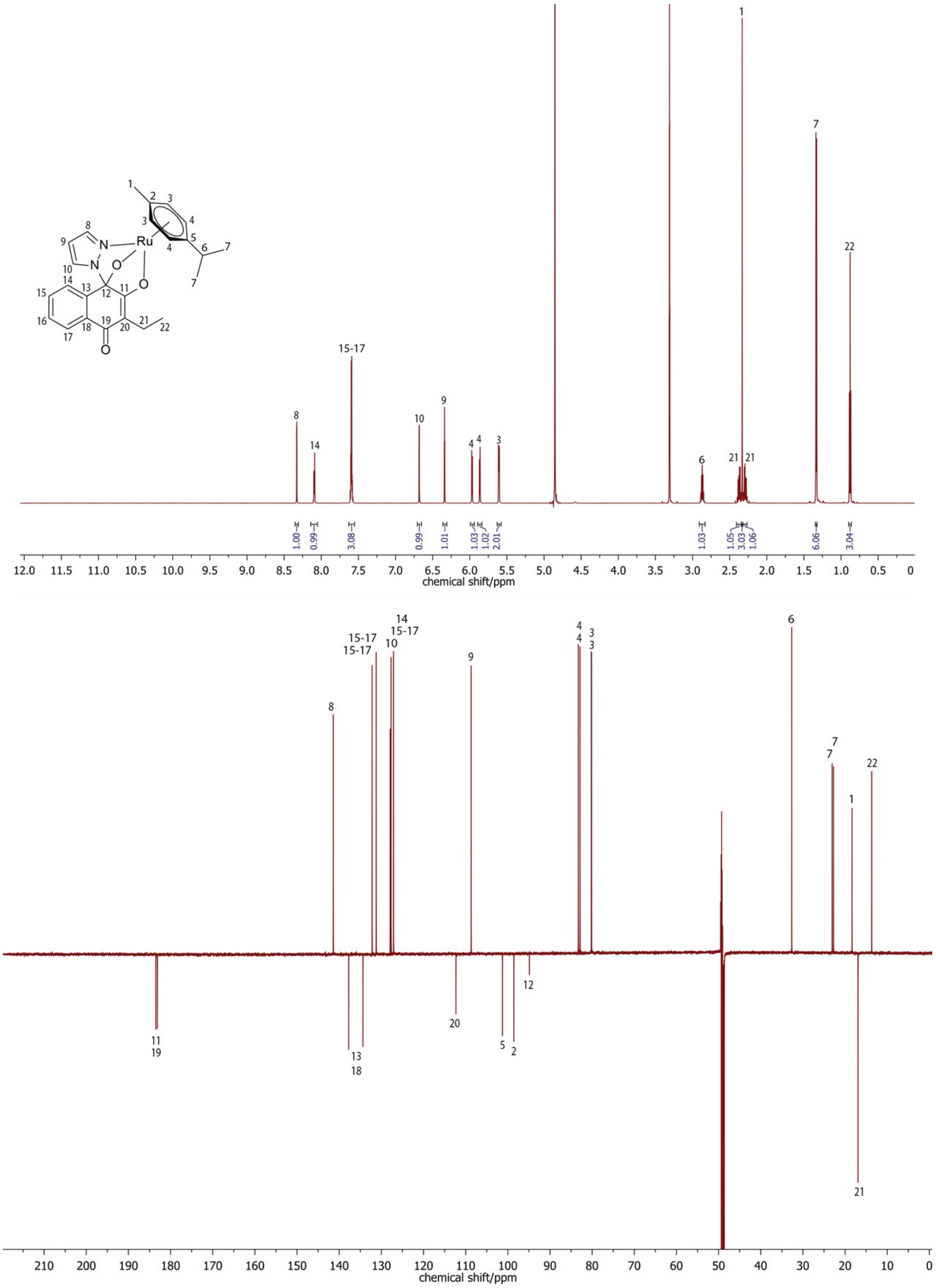

Suppl. Figure S2. Structure and atom numbering (above), ^{1}H-NMR (centre) and ^{13}C-NMR (below) of complex (**1a**) in MeOD.

[3-(Morpholinomethyl)-4-oxo-(1H-κN[2]-pyrazol-1-yl)-1,4-dihydronaphtalene-1,2-bis(olato)-κO[1]-κO[2])(η[6]-p-cymene)ruthenium(II)] (**1b**)

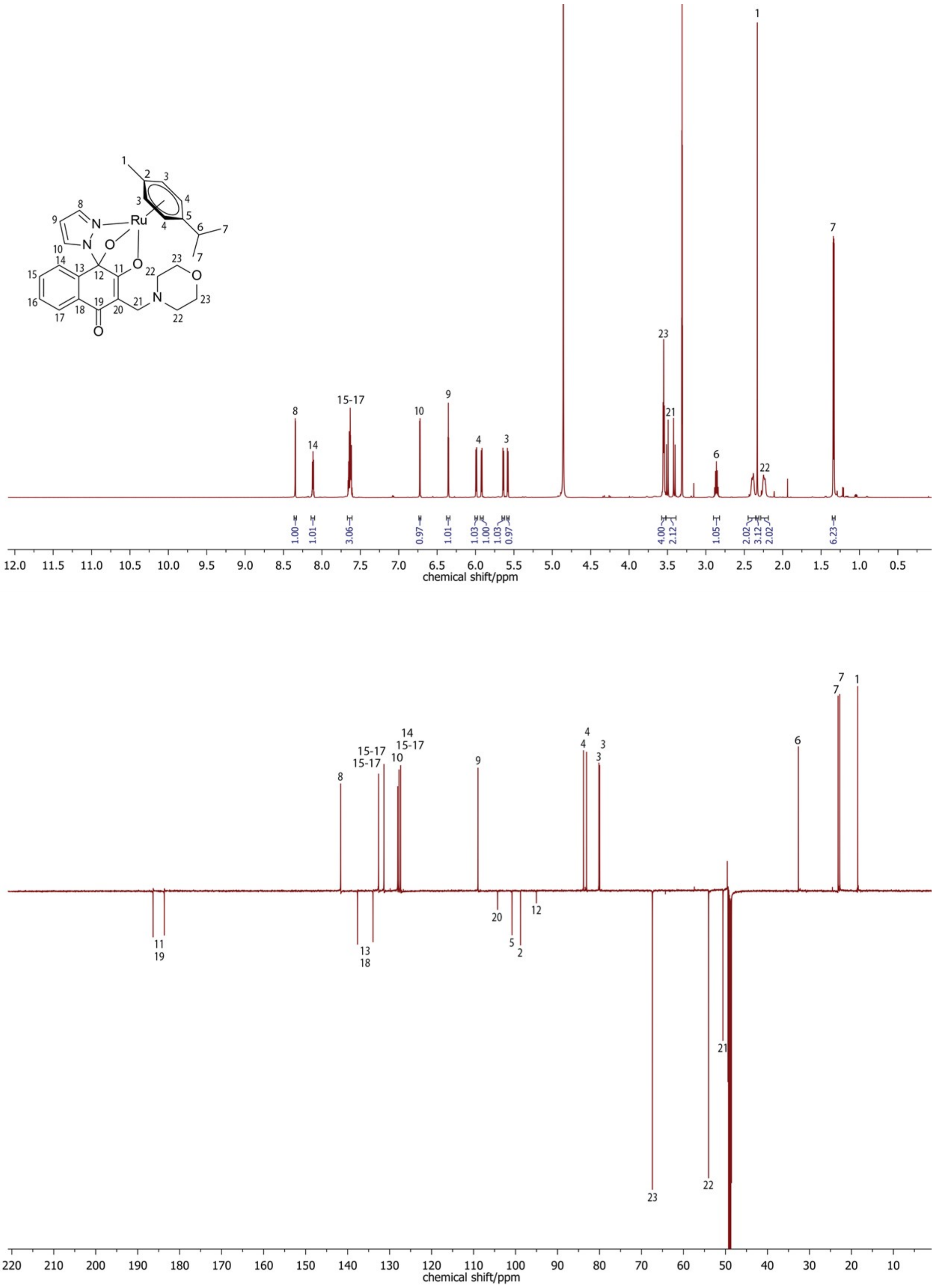

Suppl. Figure S3. Structure and atom numbering (above), ^{1}H-NMR (centre) and ^{13}C-NMR (below) of complex (**1b**) in MeOD.

[3-Ethyl-4-oxo-(1H-κN^2-pyrazol-1-yl)-1,4-dihydronaphtalene1,2-bis(olato)-κO^1-κO^2)(η^6-p-cymene)osmium(II)] (**2a**)

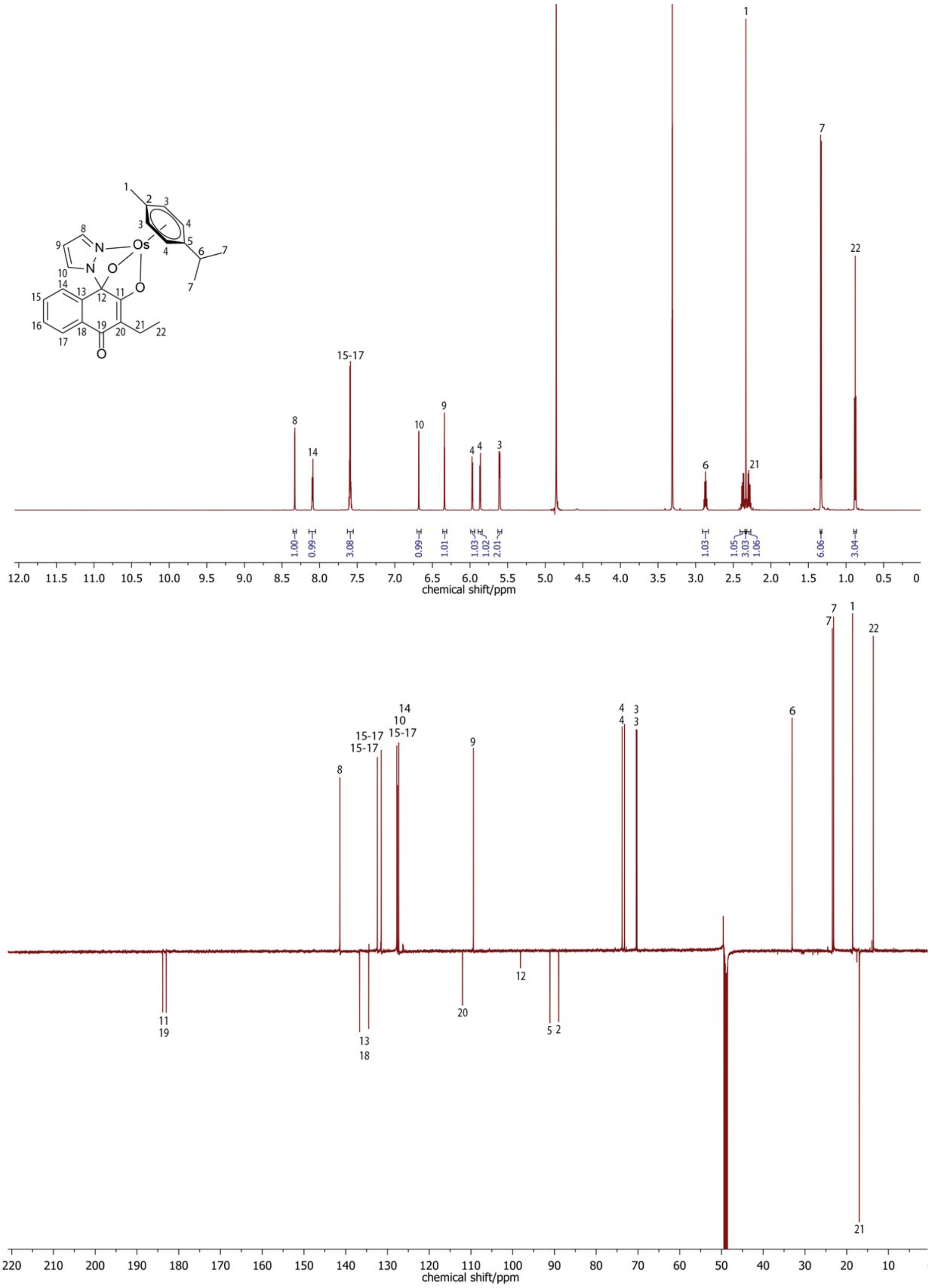

Suppl. Figure S4. Structure and atom numbering (above), ^{1}H-NMR (centre) and ^{13}C-NMR (below) of complex (**2a**) in MeOD.

[3-(Morpholinomethyl)-4-oxo-(1H-κN^2-pyrazol-1-yl)-1,4-dihydronaphtalene-1,2-bis(olato)-κO^1-κO^2)(η^6-p-cymene)osmium(II)] (**2b**)

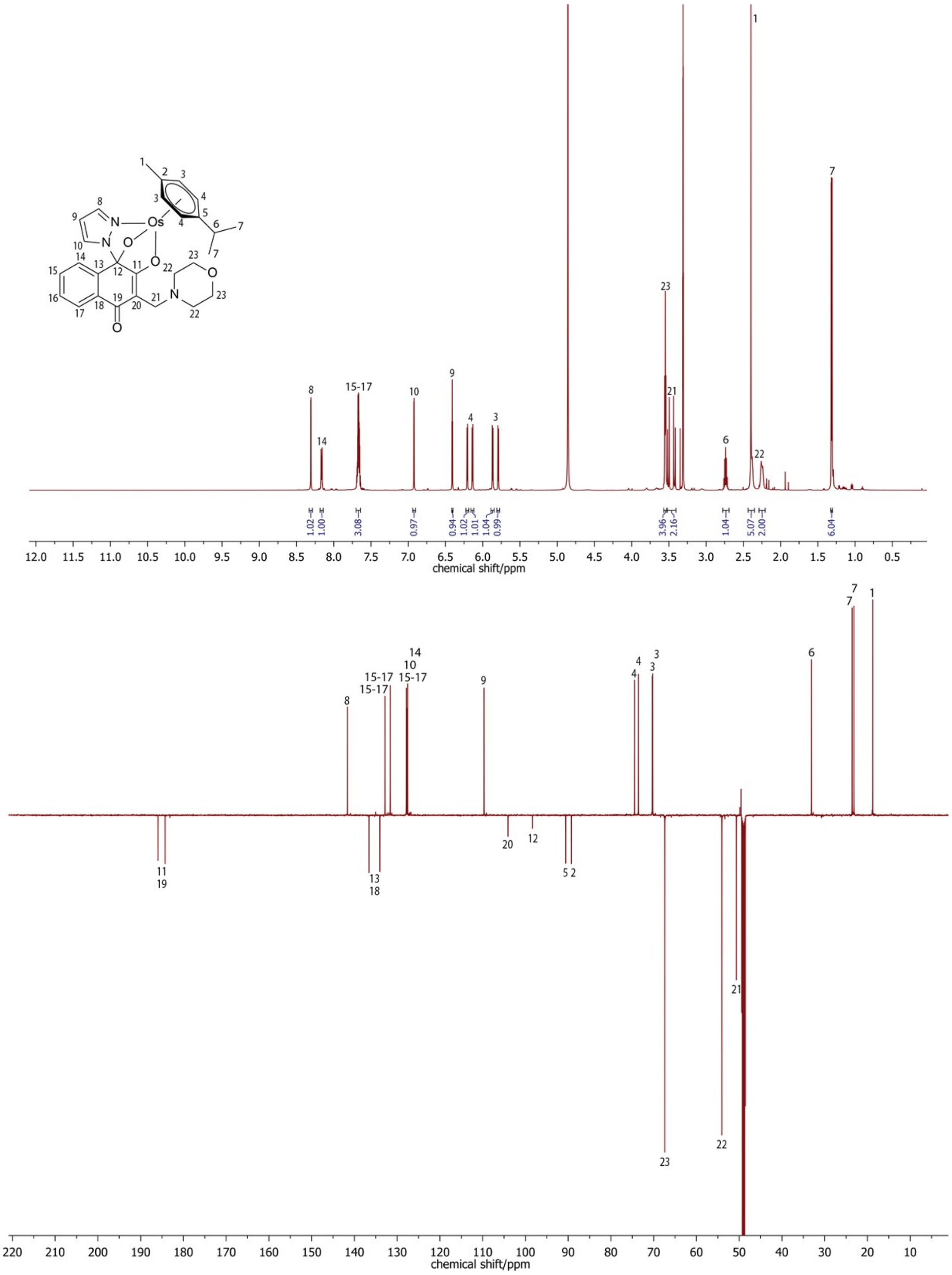

Suppl. Figure S5. Structure and atom numbering (above), ^{1}H-NMR (centre) and ^{13}C-NMR (below) of complex (**2b**) in MeOD.

Supplemental ESI-MS spectra for compound characterization

[Ethyl-4-oxo-(1H-κN^2-pyrazol-1-yl)-1,4-dihydronaphtalene1,2-bis(olato)-κO^1-κO^2)(η^6-p-cymene)ruthenium(II)] (**1a**)

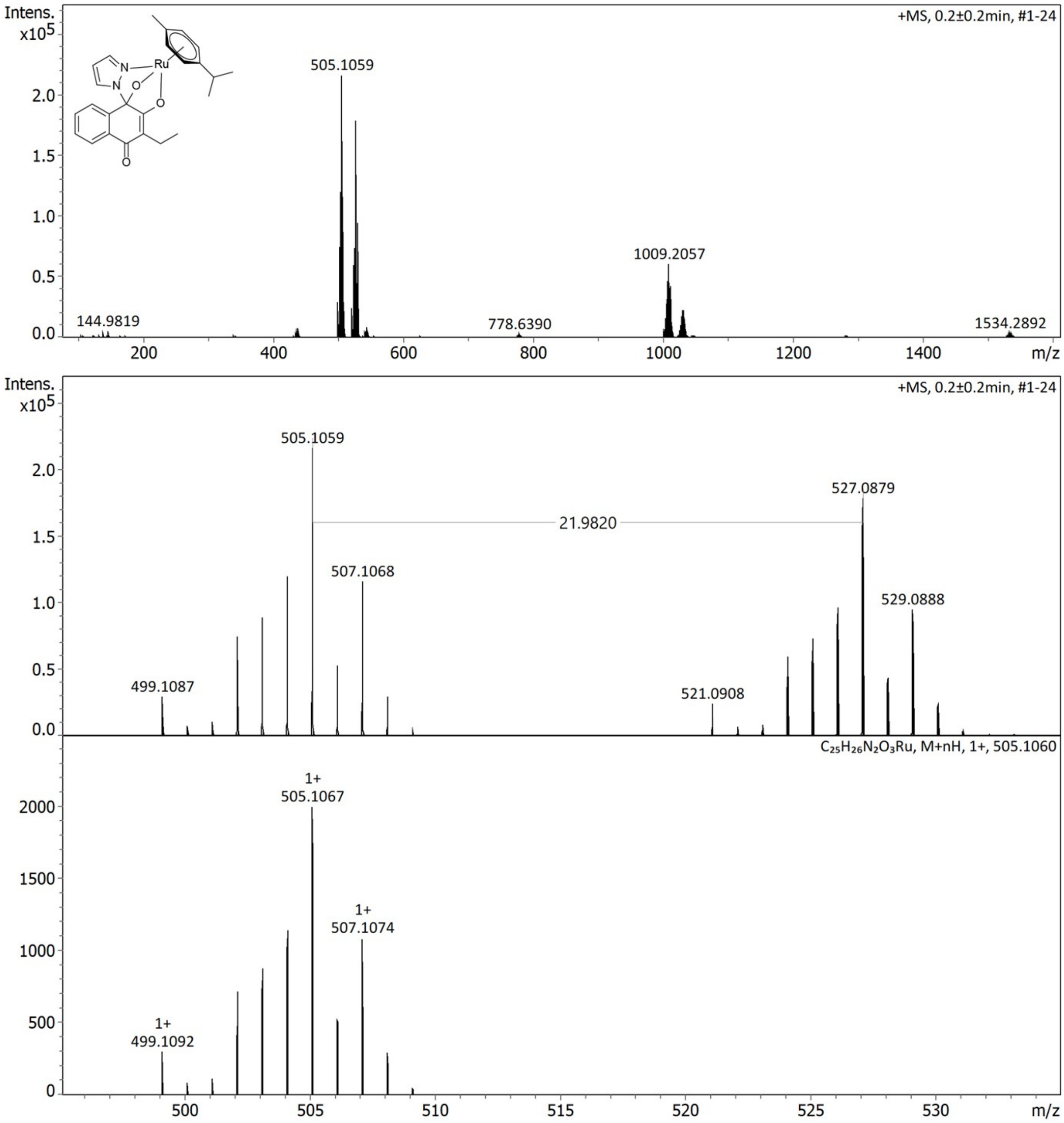

Suppl. Figure S6. HRMS spectrum of complex (**1a**).

[3-(Morpholinomethyl)-4-oxo-(1H-κN[2]-pyrazol-1-yl)-1,4-dihydronaphtalene-1,2-bis(olato)-κO[1]-κO[2])(η[6]-p-cymene)ruthenium(II)] (**1b**)

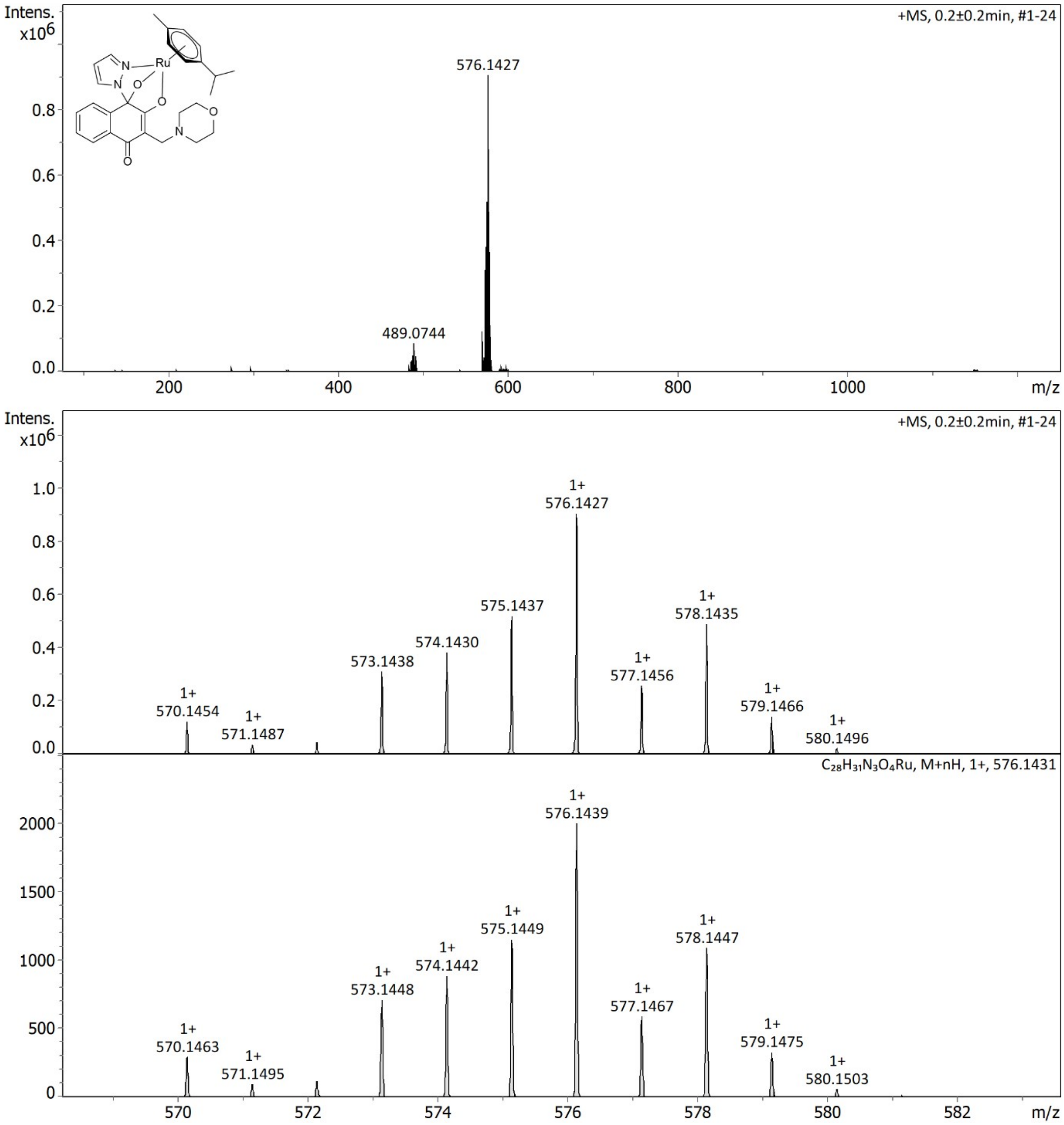

Suppl. Figure S7. HRMS spectrum of complex (**1b**).

[Ethyl-4-oxo-(1H-κN²-pyrazol-1-yl)-1,4-dihydronaphtalene1,2-bis(olato)-κO¹-κO²)(η⁶-p-cymene)osmium(II)] (**2a**)

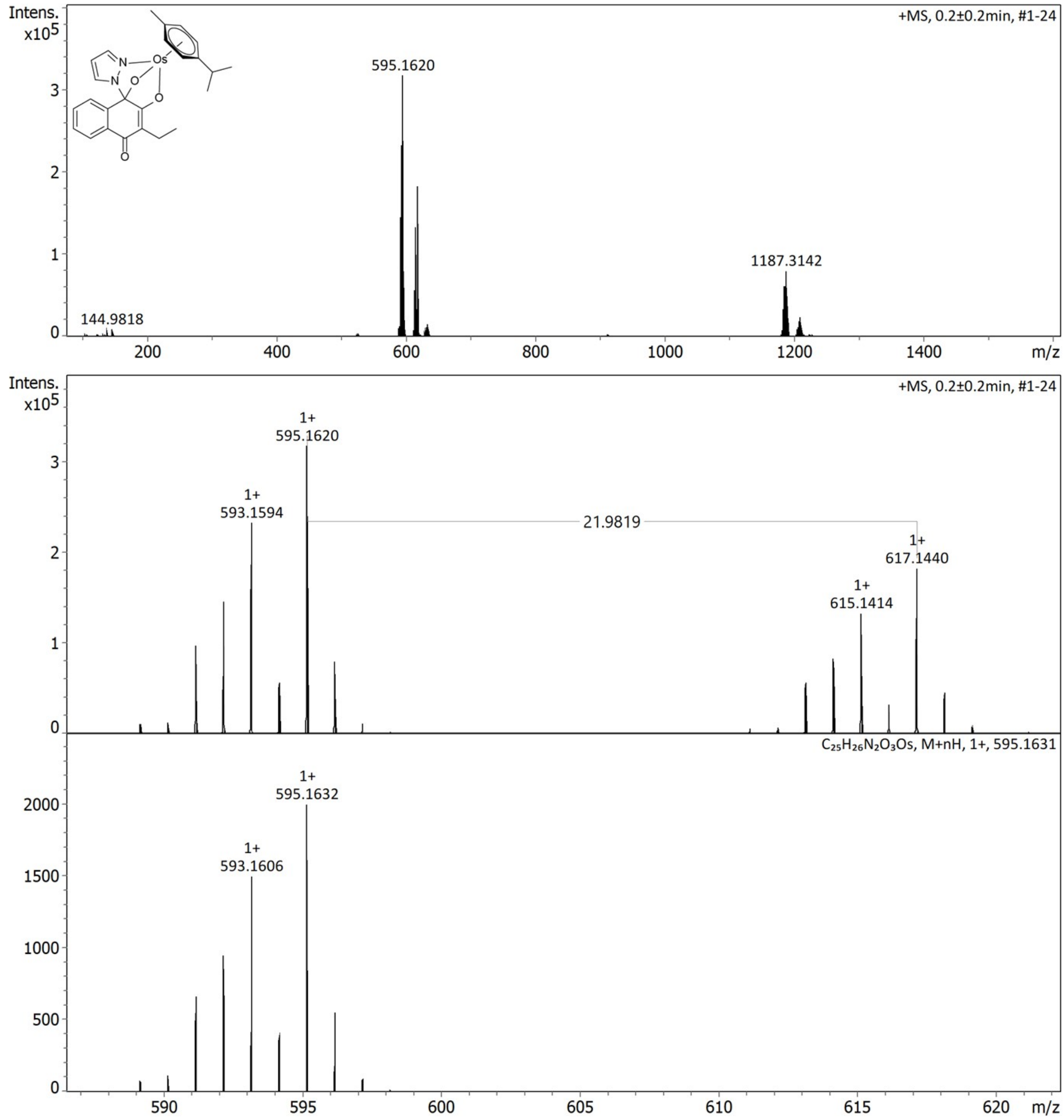

Suppl. Figure S8. HRMS spectrum of complex (**2a**).

[3-(Morpholinomethyl)-4-oxo-(1H-κN^{2}-pyrazol-1-yl)-1,4-dihydronaphtalene-1,2-bis(olato)-κO^{1}-κO^{2})(η^{6}-p-cymene)osmium(II)] (**2b**)

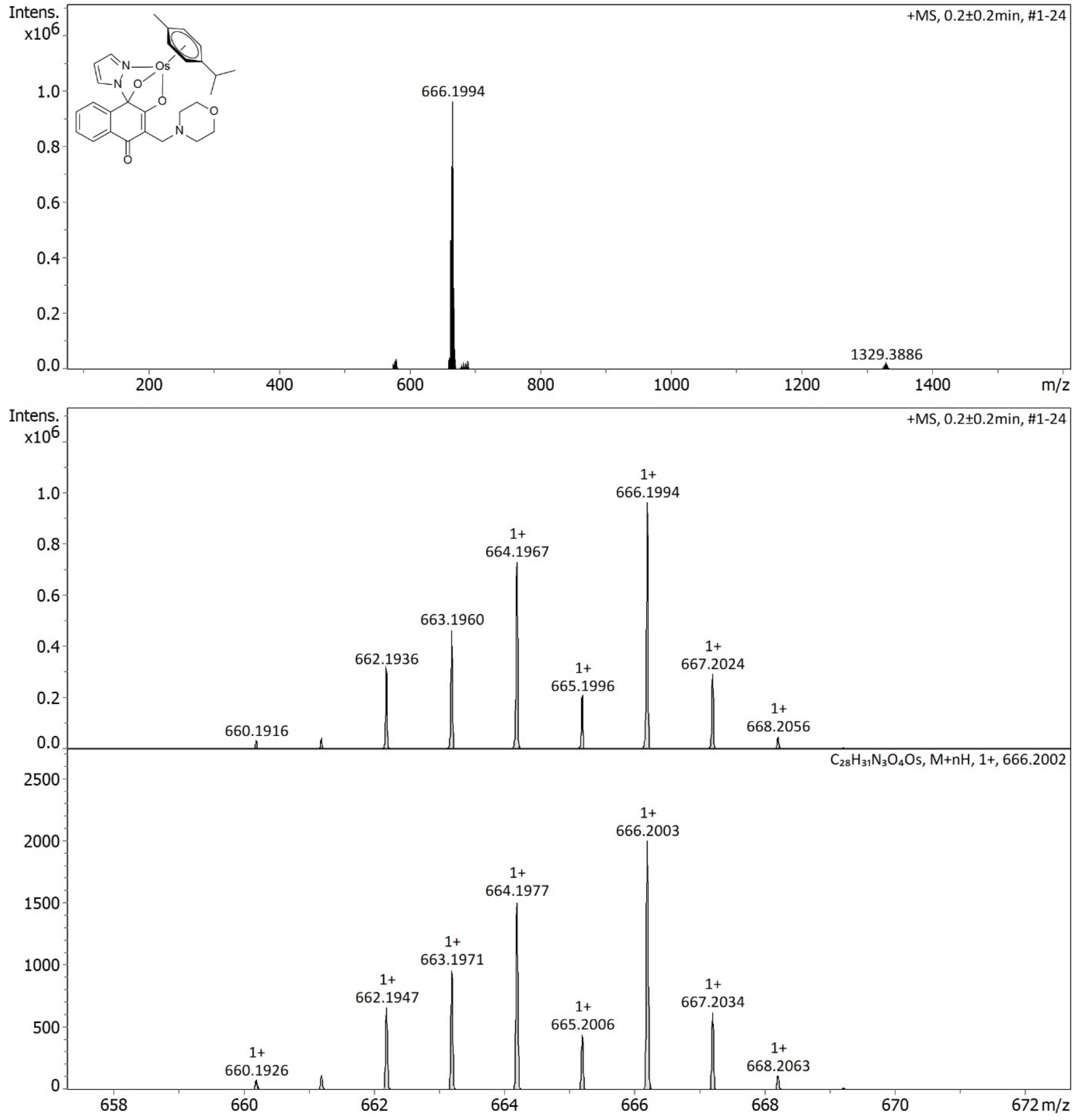

Suppl. Figure S9. HRMS spectrum of complex (**2b**).

Supplemental Figures

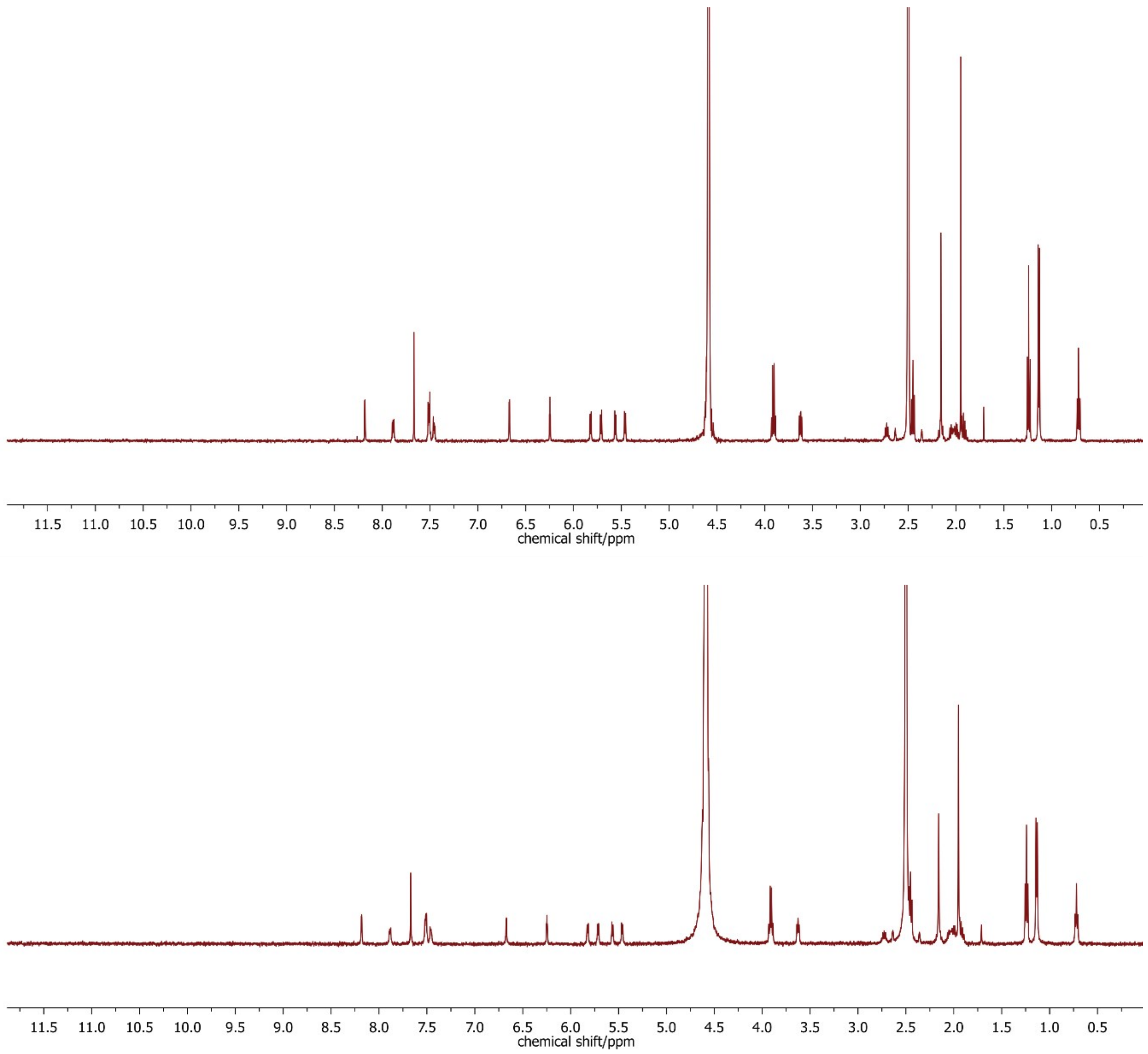

Suppl. Figure S10. Representative spectra of complex **1a**, 9-ethylguanine and methionine in D_2O/20% DMSO at 0 h (above) and after 1.5 h (below).

(A)

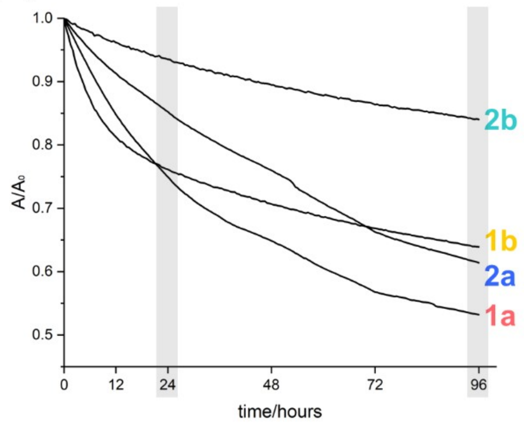

Intact complex [%(A/A0)]	24 h	96 h
2b	94	84
1b	76	64
2a	85	61
1a	75	53

(B)

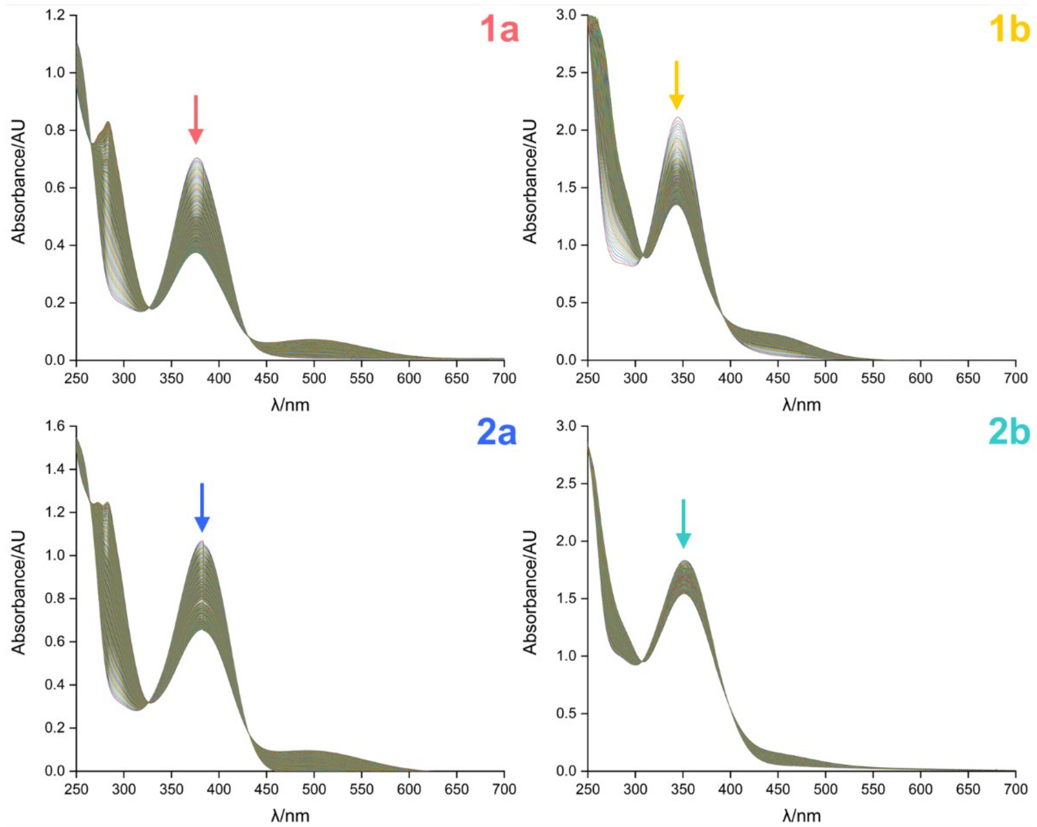

Suppl. Figure S11. UV-Vis stability studies of **1a**/**2a** at 40 µM and **1b**/**2b** at 80 µM in PBS (1% DMSO) over the course of 96 h. UV-Vis spectra were acquired every 30 min over the entire incubation period. (**A**) Time-dependent percentage of the remaining intact compound at the absorption maximum of around 350–370 nm with 24 h and 96 h highlighted. (**B**) Individual kinetic UV-Vis spectra over 96 h.

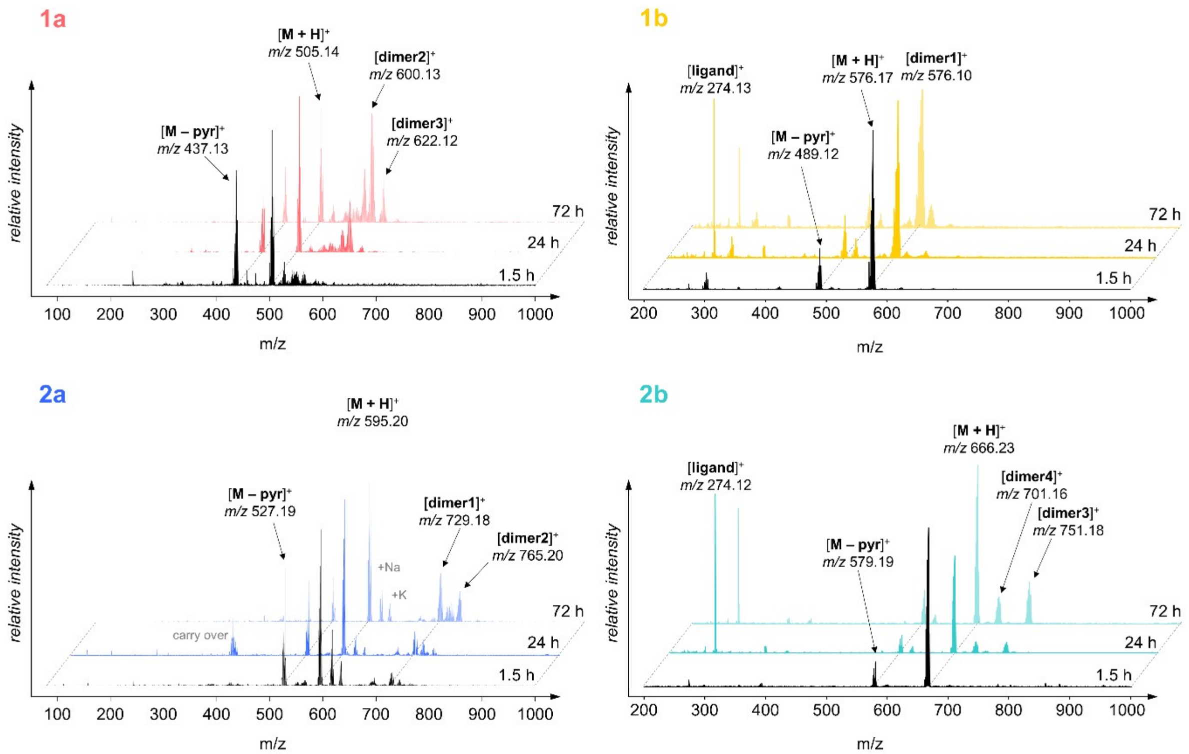

Suppl. Figure S12. ESI-MS spectra of the incubation of the four metal(arene) compounds in aqueous solution. Mass spectra were acquired after 1.5 h, 24 h and 72 h. Identified mass signals are annotated. Dimers refer to different dinuclear metal(arenes) with diverse bridging ligands, including pyrazole.

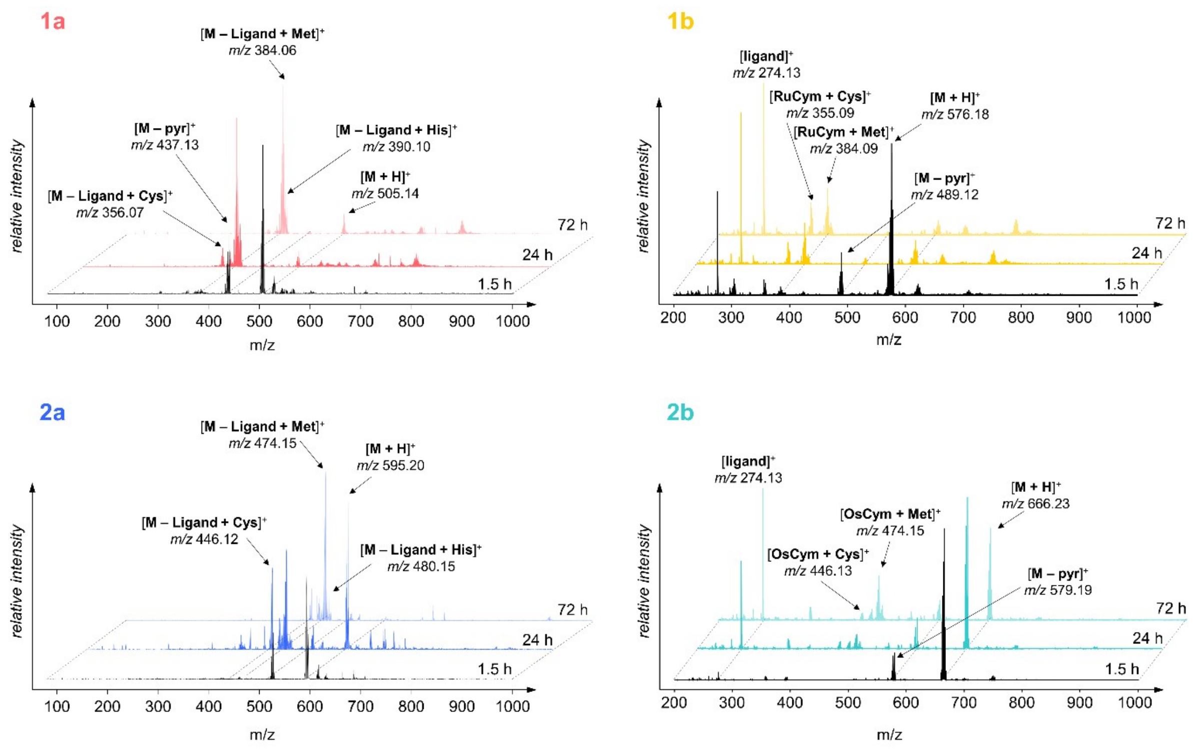

Suppl. Figure S13. ESI-MS spectra of the incubation of the four metal(arene) compounds of this study at equimolar concentration with a mixture of cysteine (Cys), methionine (Met), histidine (His), adenosine triphosphate (ATP) and guanosine triphosphate (GTP). Mass spectra were acquired after 1.5 h, 24 h and 72 h. Identified mass signals are annotated.

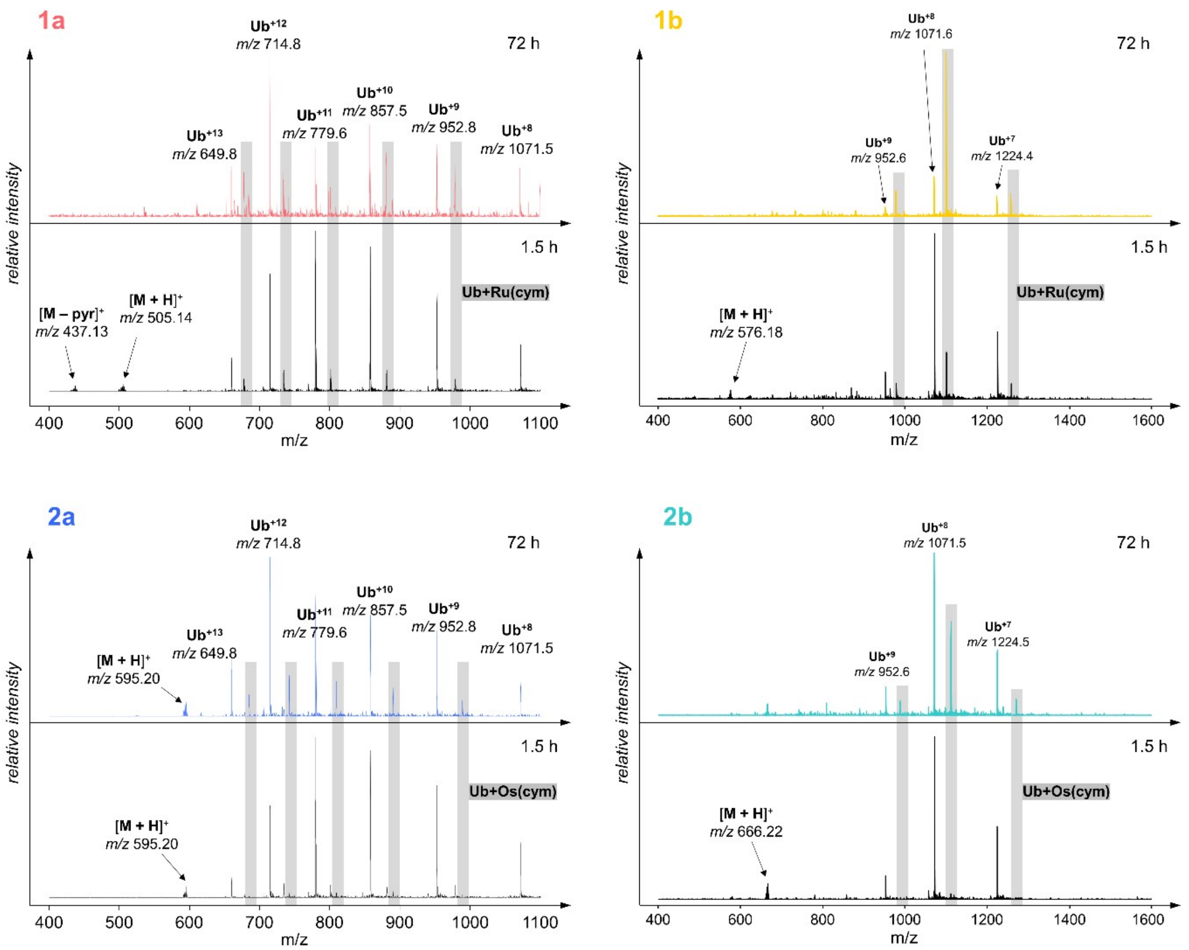

Suppl. Figure S14. ESI-MS spectra of the incubation of the four metal(arene) compounds of this study with an equimolar concentration of ubiquitin. Mass spectra are shown after 1.5 h and 72 h of incubation and metal(arene) adducts are highlighted in grey background. Intact compounds were observed after 1.5 h.

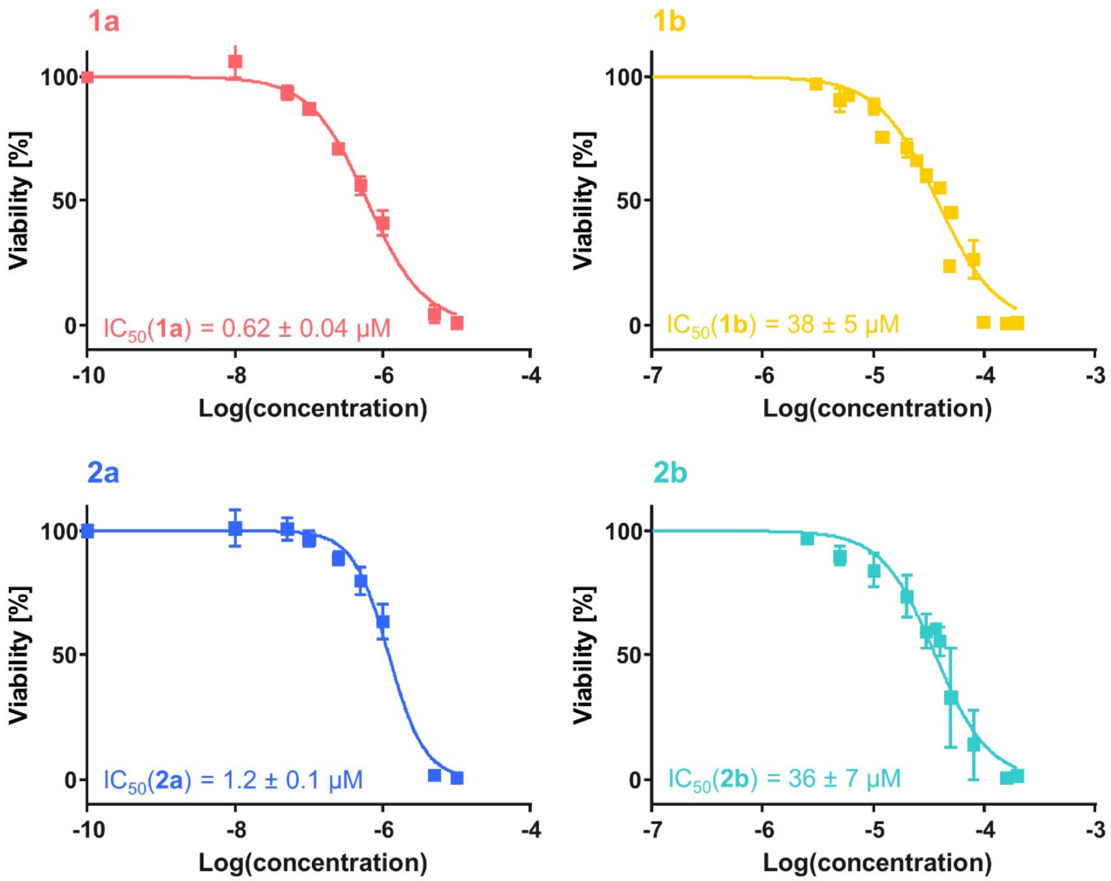

Suppl. Figure S15. Concentration-effect curves of **1a**, **1b**, **2a** and **2b** after a 24 h incubation of SW480 colon carcinoma cells. A total of 4000 cells were seeded per well for experiments with **1a** and **2a** and a total of 10'000 cells were seeded per well for experiments with **1b** and **2b**. The IC_{50} values are presented as the mean ± standard deviation of at least three independent experiments, each in at least three replicates.

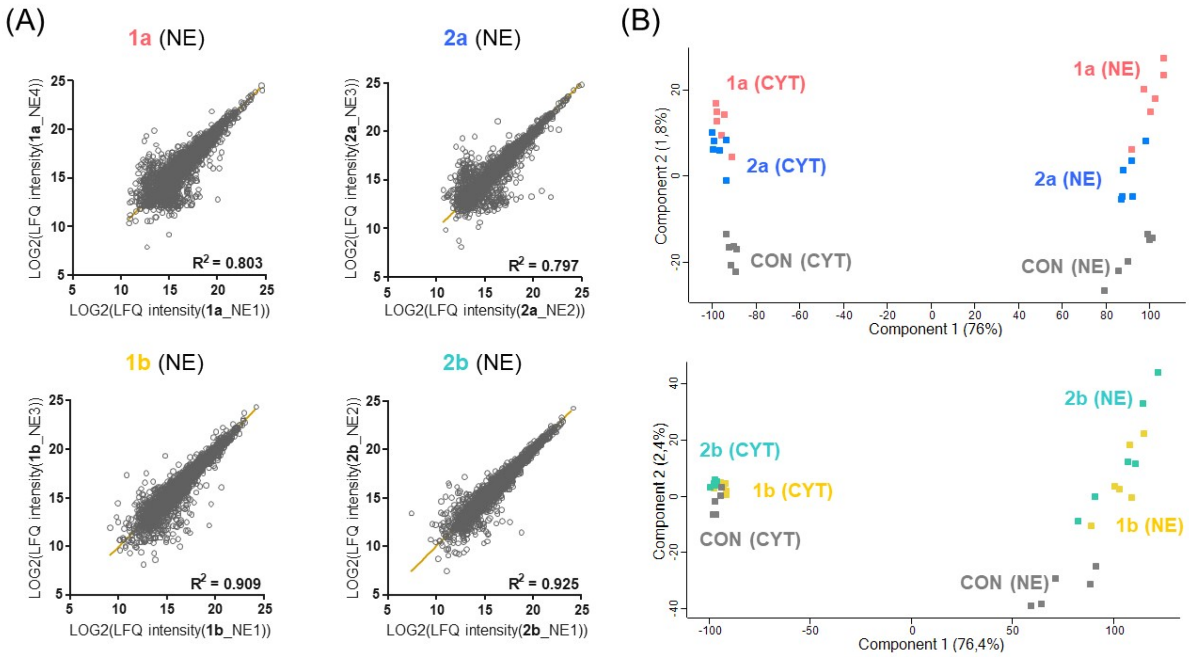

Suppl. Figure S16. Qualification of the proteomic profiling experiments. (**A**) Scatter plots show the precision of two representative replicates (out of six) of nuclear extracts (NE) representing the last step of fractionation. (**B**) Perturbations with **1a**/**2a** and **1b**/**2b** were carried out against their own solvent-treated controls (CON). The principle component analysis shows the pairs **1a**/**2a** and **1b**/**2b** against their respective controls. Each square represents a sample of either cytoplasmic (CYT) or NE fractions.

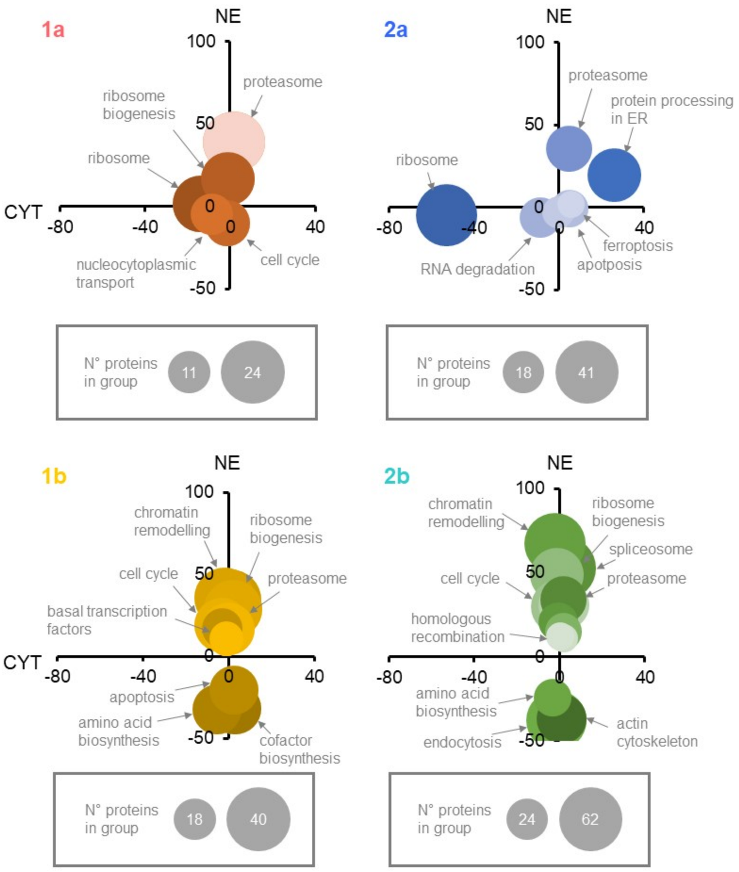

Suppl. Figure S17. (**A**) Regulomes showing KEGG pathways affected by the treatment of in SW480 cancer cells with the four compounds. The axes depict the cytoplasmic (CYT) and nuclear (NE) subcellular space.

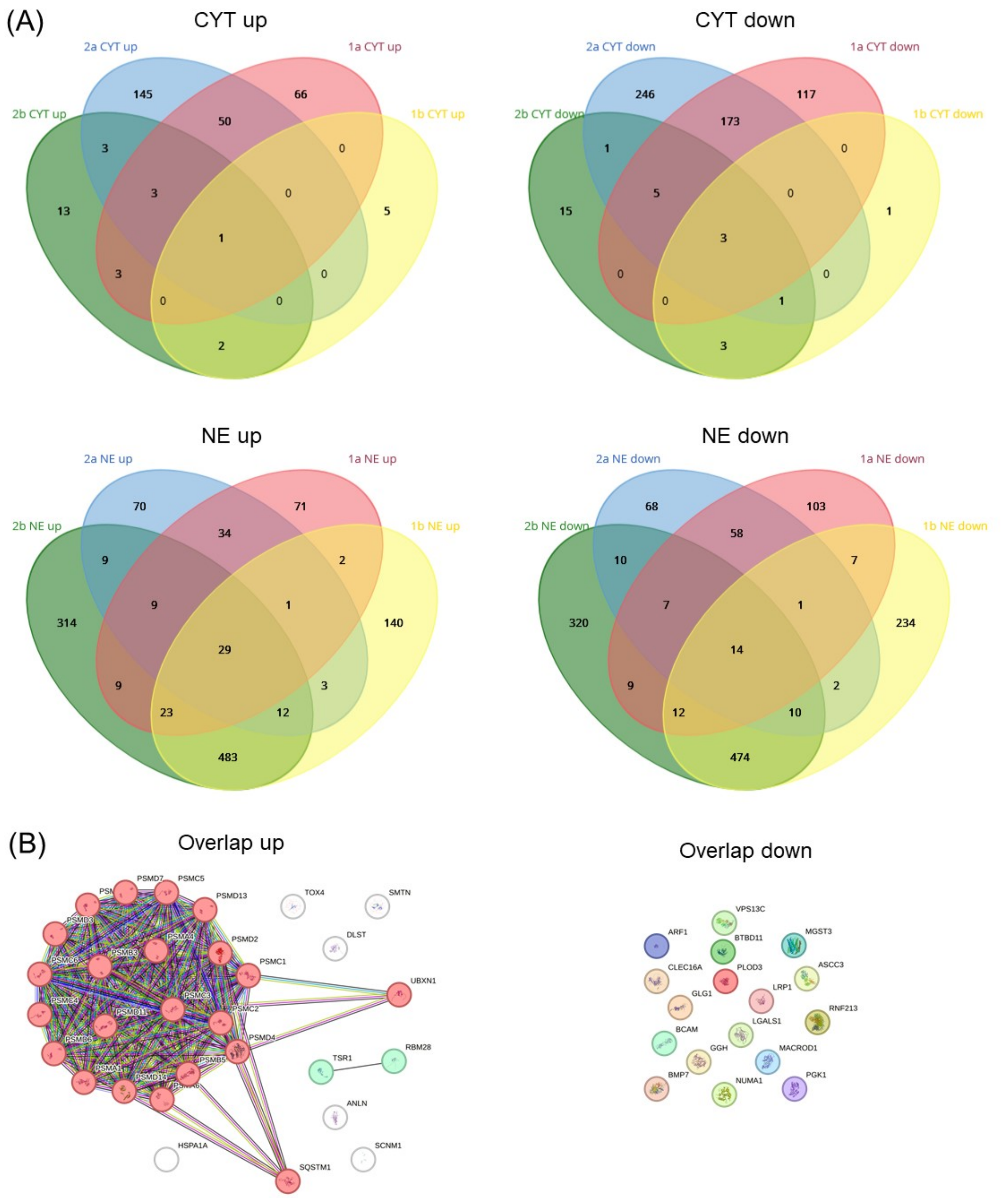

Suppl. Figure S18. (**A**) VENN diagrams showing the shared significantly proteins of each treatment according to cytoplasmic (CYT) and nuclear (NE) fractions and direction of regulation. up = up-regulated; down = down-regulated. (**B**) STRING network of the shared proteins of all four treatments according to upregulated (*left*) and down-regulated (*right*) proteins.

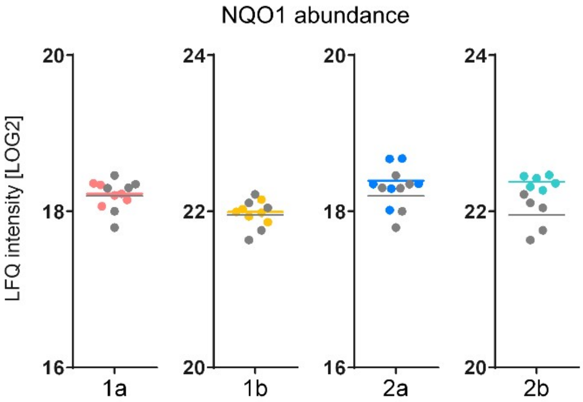

Suppl. Figure S19. Regulation of NQO1, the target of hydroxy-naphthoquinones, in SW480 cancer cells upon treatment with the compounds of this study after 24 h. Control samples are shown in grey and the treated samples are coloured.

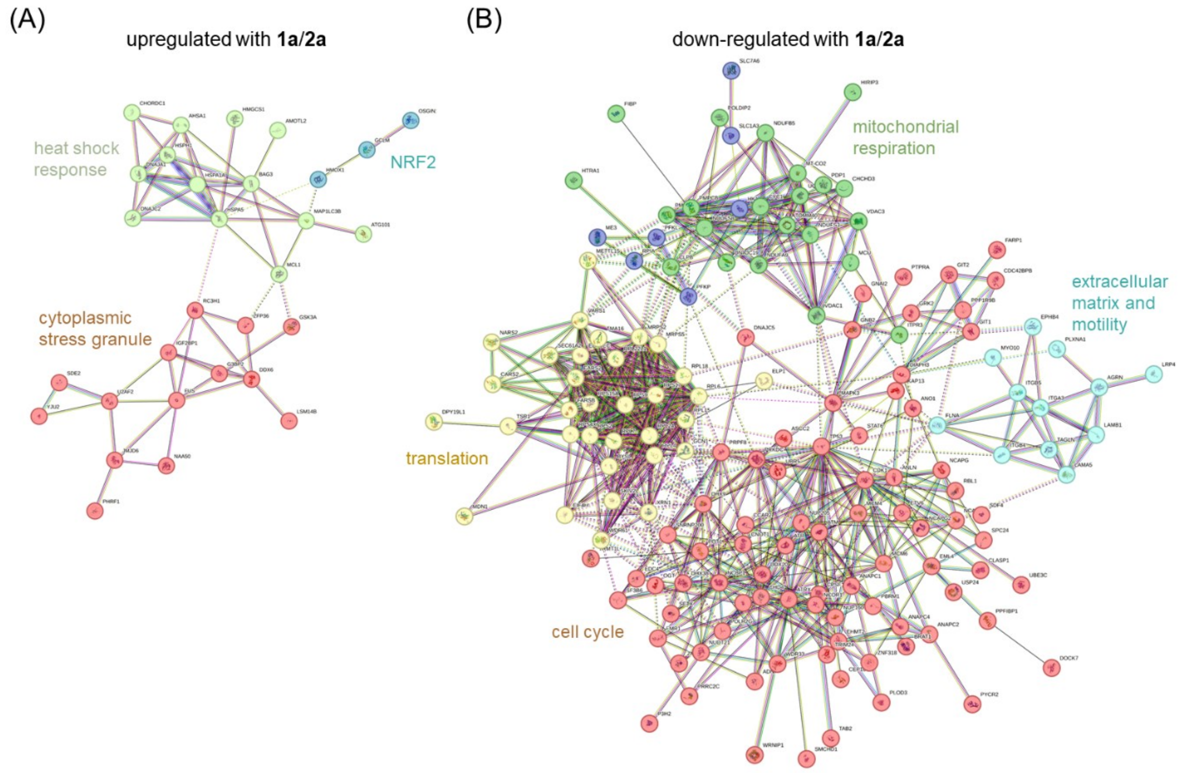

Suppl. Figure S20. (**A**) STRING network analysis of the shared upregulated proteins of **1a** and **2a** treated SW480 cancer cells in both the CYT and NE fractions. (**B**) STRING network analysis of the shared down-regulated proteins of **1a** and **2a** treated SW480 cancer cells in both the CYT and NE fractions.

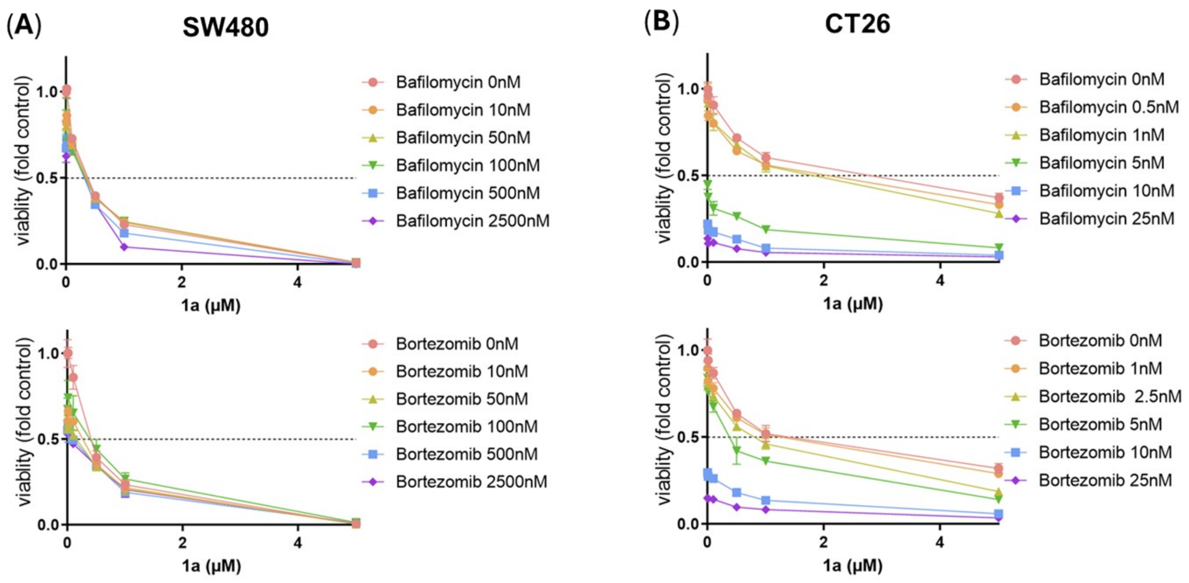

Suppl. Figure S21. Dose-response curves of **1a** in combination with bafilomycin or bortezomib after 24 h incubation of (**A**) SW480 or (**B**) CT26 cells. One representative of at least three independent experiments is shown.

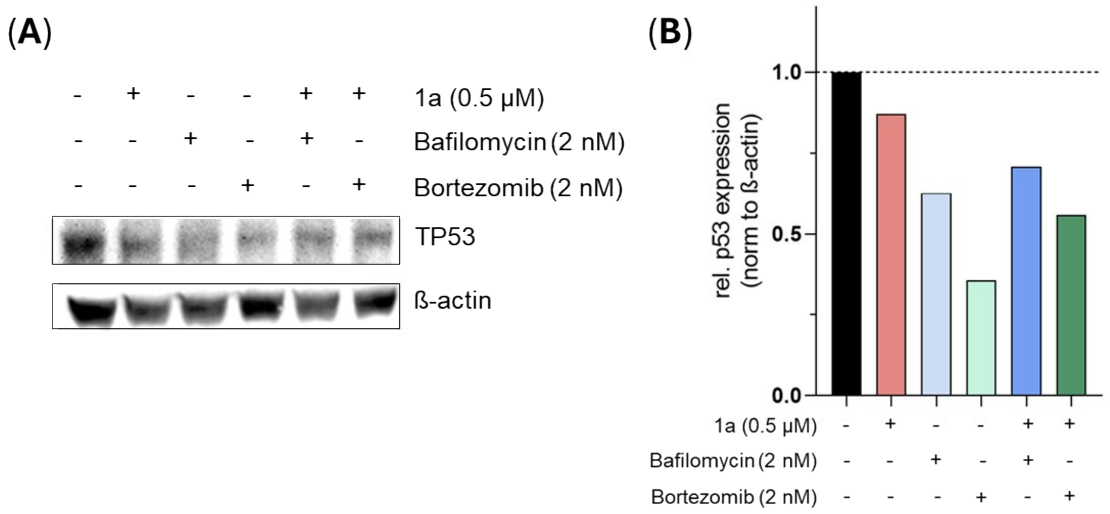

Suppl. Figure S22. (A) Protein expression levels of TP53 in whole cell lysates of CT26 cells treated with indicated compounds for 24 h and analysed by Western blotting. β-actin served as loading control. (**B**) TP53 protein expression relative to DMSO control, normalized to ß-actin quantified from (**A**) using Image J.

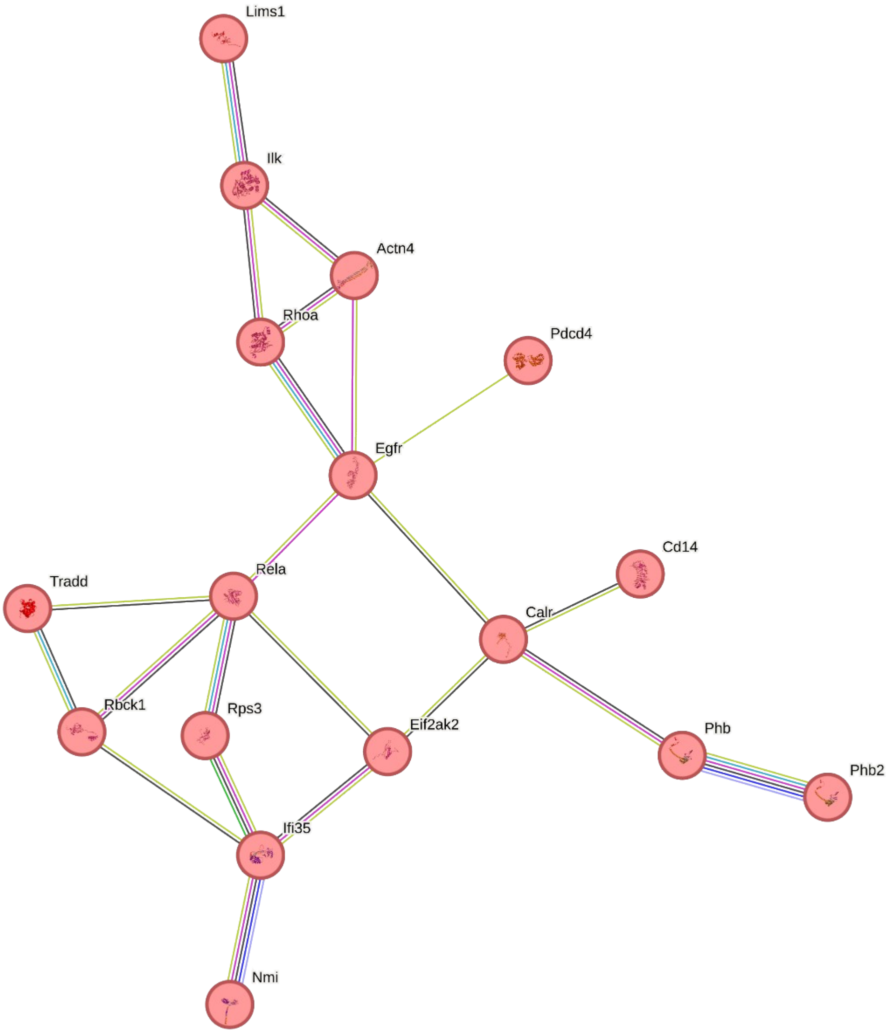

Suppl. Figure S23. STRING network analysis of the down-regulated proteins in tumour tissue of CT26-bearing mice after treatment with **1a** over five days at 10 mg·kg^{-1} i.p.

Supplementary Tables

Table S1. Concentrations to inhibit 50% of cell growth (IC_{50}) in micromolar concentrations. IC50-values are reported after a specific incubation time. IC_{50} values of incubation times of 96 h were obtained from Ref. 1. **a** = 3Et-NQ, **b** = 3Morph-NQ.

Compound	IC_{50} values [µM]		
	**SW480 24h**	**SW480 96h[1]**	**CH1/PA-1 96h[1]**
**cisplatin**	n.d.	2.3 ± 0.2	0.073± 0.001
**a**	n.d.	100 ± 10	173 ± 10
**b**	n.d.	160 ± 30	>200
**[Ru(cym)(3Et-NQ)Cl]**	n.d.	27 ± 1	70 ± 10
**1a**	0.62 ± 0.04	0.046 ± 0.007	62 ± 5
**1b**	38 ± 5	33 ± 2	150 ± 20
**2a**	1.2 ± 0.1	n.d.	n.d.
**2b**	36 ± 7	n.d.	n.d.

Table S2. Number of significantly regulated proteins in the cytoplasmic (CYT) and nuclear (NE) fractions of compound-treated SW480 cancer cells with respect to solvent-treated controls. Each condition was carried out in hexuplicates. A total of 5657 proteins were identified overall.

Compound	CYT		NE		Total
	N° proteins upregulated	N° proteins down-regulated	N° proteins upregulated	N° proteins down-regulated	
1a	123	298	178	211	810
2a	202	429	167	170	968
1b	8	8	710	772	1498
2b	25	28	896	877	1826

Table S3. Results of gene set variation analysis (GSVA) of in vivo proteomic data with respect to terms according to gene ontology biological processes (GO BP). Statistical significance of term enrichments was calculated using a Benjamini-Hochberg false discovery rate (adjusted P-Value). FC = Fold-change

Gene Set Name	N° Proteins in set	Log2(FC)	adj P-Value
**Tumour**			
GOBP REGULATION OF INTRINSIC APOPTOTIC SIGNALING PATHWAY IN RESPONSE TO OSMOTIC STRESS	3	0,70	2,7E-02
GOBP REGULATION OF SMOOTHENED SIGNALING PATHWAY	8	0,70	2,3E-02
GOBP CELLULAR RESPONSE TO ARSENIC CONTAINING SUBSTANCE	7	0,48	1,6E-02
GOBP SMOOTHENED SIGNALING PATHWAY	13	0,47	2,7E-02
GOBP POSITIVE REGULATION OF NIK NF KAPPAB SIGNALING	18	-0,47	1,2E-02
GOBP FATTY ACID DERIVATIVE CATABOLIC PROCESS	7	-0,56	2,7E-02
GOBP CELLULAR RESPONSE TO LIPOPROTEIN PARTICLE STIMULUS	10	-0,65	2,3E-02
**Plasma**			
GOBP REGULATION OF RESPONSE TO ENDOPLASMIC RETICULUM STRESS	2	1,14	1,1E-03
GOBP LYMPHOCYTE CHEMOTAXIS	2	1,13	1,4E-03
GOBP POSITIVE REGULATION OF INTERLEUKIN 1 PRODUCTION	3	1,03	4,5E-03
GOBP POSITIVE REGULATION OF ADAPTIVE IMMUNE RESPONSE	4	0,76	2,6E-02
**Liver**			
GOBP LEUKOCYTE MIGRATION INVOLVED IN INFLAMMATORY RESPONSE	2	1,02	3,2E-03
GOBP LEUKOCYTE AGGREGATION	3	0,91	2,4E-03
GOBP REGULATION OF MONOCYTE DIFFERENTIATION	2	0,70	4,5E-02
GOBP ENDOPLASMIC RETICULUM TUBULAR NETWORK ORGANIZATION	5	0,65	4,1E-02
GOBP POSITIVE REGULATION OF TOR SIGNALING	7	0,55	4,5E-02
GOBP REGULATION OF MITOCHONDRIAL FISSION	6	0,54	3,8E-02
GOBP MONOCYTE ACTIVATION	3	0,54	3,0E-02
GOBP RESPONSE TO AMINO ACID STARVATION	8	0,51	4,4E-02
GOBP RESPONSE TO INTERLEUKIN 1	15	0,43	1,4E-02
GOBP ENDOPLASMIC RETICULUM ORGANIZATION	35	0,39	4,3E-02
GOBP TRANSITION METAL ION TRANSPORT	22	0,38	4,8E-02
GOBP TOR SIGNALING	16	0,37	4,4E-02
GOBP NADH DEHYDROGENASE COMPLEX ASSEMBLY	30	-0,55	2,7E-03
GOBP OXIDATIVE PHOSPHORYLATION	70	-0,55	2,0E-03
GOBP AEROBIC RESPIRATION	94	-0,56	1,5E-03
GOBP ATP SYNTHESIS COUPLED ELECTRON TRANSPORT	47	-0,60	5,2E-04
GOBP MITOCHONDRIAL ELECTRON TRANSPORT NADH TO UBIQUINONE	32	-0,61	7,5E-04

References

1. H. Geisler, J. Westermayr, K. Cseh, D. Wenisch, V. Fuchs, S. Harringer, S. Plutzar, N. Gajic, M. Hejl, M. A. Jakupec, P. Marquetand and W. Kandioller, Tridentate 3-substituted naphthoquinone ruthenium arene complexes: Synthesis, characterization, aqueous behavior, and theoretical and biological studies, *Inorg. Chem.*, 2021, **60**, 9805-9819.
2. H. Geisler, S. Harringer, D. Wenisch, R. Urban, M. A. Jakupec, W. Kandioller and B. K. Keppler, Systematic study on the cytotoxic potency of commonly used dimeric metal precursors in human cancer cell lines, *ChemistryOpen*, 2022, **11**, e202200019.
3. B. Neuditschko, A. P. King, Z. Huang, L. Janker, A. Bileck, Y. Borutzki, S. C. Marker, C. Gerner, J. J. Wilson and S. M. Meier-Menches, An anticancer rhenium tricarbonyl targets Fe−S cluster biogenesis in ovarian cancer cells, *Angew. Chem., Int. Ed.*, 2022, **61**, e202209136.
4. A. Zougman, P. J. Selby and R. E. Banks, Suspension trapping (STrap) sample preparation method for bottom-up proteomics analysis, *Proteomics*, 2014, **14**, 1006-1000.
5. W. Berger, L. Elbling and M. Micksche, Expression of the major vault protein LRP in human non-small-cell lung cancer cells: activation by short-term exposure to antineoplastic drugs, *Int. J. Cancer*, 2000, **88**, 293-300.
6. A. T. Feldman and D. Wolfe, Tissue processing and hematoxylin and eosin staining, *Methods Mol. Biol.*, 2014, **1180**, 31-43.
7. P. Bortel, G. Hagn, L. Skos, A. Bileck, V. Paulitschke, P. Paulitschke, L. Gleiter, T. Mohr, C. Gerner and S. M. Meier-Menches, Memory effects of prior subculture may impact the quality of multiomic perturbation profiles, *Proc. Natl. Acad. Sci.*, 2024, **121**, e2313851121.
8. L. Skos, C. Schmidt, S. R. Thomas, M. Park, V. Geiger, D. Wenisch, R. Bonsignore, G. Del Favero, T. Mohr, A. Bileck, C. Gerner, A. Casini and S. M. Meier-Menches, Gold-templated covalent targeting of the CysSec-dyad of thioredoxin reductase 1 in cancer cells, *Cell Rep. Phys. Sci.*, 2024, **5**, 102072.
9. T. Weiss, S. Taschner-Mandl, L. Janker, A. Bileck, F. Rifatbegovic, F. Kromp, H. Sorger, M. O. Kauer, C. Frech, R. Windhager, C. Gerner, P. F. Ambros and I. M. Ambros, Schwann cell plasticity regulates neuroblastic tumor cell differentiation via epidermal growth factor-like protein 8, *Nat. Commun.*, 2021, **12**, 1624.
10. J. Cox and M. Mann, MaxQuant enables high peptide identification rates, individualized p.p.b.-range mass accuracies and proteome-wide protein quantification, *Nat. Biotechnol.*, 2008, **26**, 1367-1372.
11. A. Bateman, M.-J. Martin, S. Orchard, M. Magrane, R. Agivetova, S. Ahmad, E. Alpi, E. H. Bowler-Barnett, R. Britto, B. Bursteinas*, et al.*, UniProt: the universal protein knowledgebase in 2021, *Nucleic Acids Res.*, 2021, **49**, D480-D489.
12. S. Tyanova, T. Temu, P. Sinitcyn, A. Carlson, M. Y. Hein, T. Geiger, M. Mann and J. Cox, The Perseus computational platform for comprehensive analysis of (prote)omics data, *Nat. Methods*, 2016, **13**, 731-740.
13. B. T. Sherman, M. Hao, J. Qiu, X. Jiao, M. W. Baseler, H. C. Lane, T. Imamichi and W. Chang, DAVID: a web server for functional enrichment analysis and functional annotation of gene lists (2021 update), *Nucleic Acids Res.*, 2022, **50**, W216-W221.
14. D. Szklarczyk, R. Kirsch, M. Koutrouli, K. Nastou, F. Mehryary, R. Hachilif, A. L. Gable, T. Fang, Nadezhda T. Doncheva, S. Pyysalo, P. Bork, Lars J. Jensen and C. von Mering, The STRING database in 2023: protein–protein association networks and functional enrichment analyses for any sequenced genome of interest, *Nucleic Acids Res.*, 2023, **51**, D638-D646.
15. M. E. Ritchie, B. Phipson, D. Wu, Y. Hu, C. W. Law, W. Shi and G. K. Smyth, LIMMA powers differential expression analyses for RNA-sequencing and microarray studies, *Nucleic Acids Res.*, 2015, **43**, e47-e47.
16. Y. Hochberg and Y. Benjamini, More powerful procedures for multiple significance testing, *Stat. Med.*, 1990, **9**, 811-818.
17. S. Hänzelmann, R. Castelo and J. Guinney, GSVA: Gene set variation analysis for microarray and RNA-Seq data, *BMC Bioinformatics*, 2013, **14**, 7.
18. A. Liberzon, A. Subramanian, R. Pinchback, H. Thorvaldsdóttir, P. Tamayo and J. P. Mesirov, Molecular signatures database (MSigDB) 3.0, *Bioinformatics*, 2011, **27**, 1739-1740.
